# Catalytic Upgrading of a Mixed Hydroxy Acid Feedstock Derived from Kraft Black Liquor

**DOI:** 10.1021/acssuschemeng.4c00212

**Published:** 2024-06-03

**Authors:** Opeyemi A. Ojelade, Qiang Fu, Sankar Nair, Christopher W. Jones

**Affiliations:** School of Chemical & Biomolecular Engineering, Georgia Institute of Technology, Atlanta, Georgia 30332-0100, United States

**Keywords:** hydrogenolysis, hydrodeoxygenation, kraft black liquor, hydroxy acids, bifunctional catalyst, carboxylic acids

## Abstract

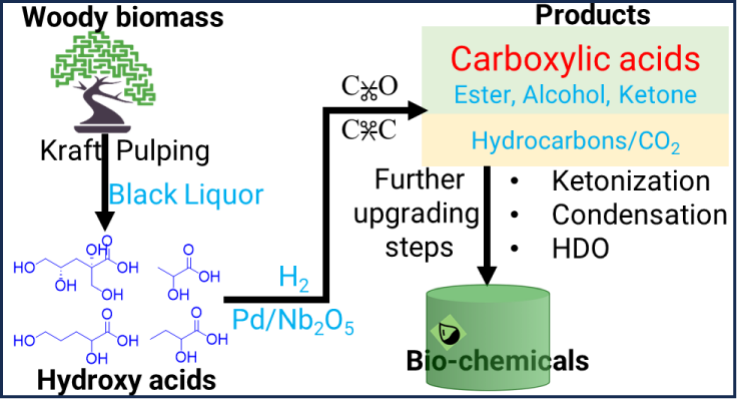

Lignocellulosic feedstocks are widely studied for sustainable liquid fuel and chemical production. The pulp and paper industry generates large amounts of kraft black liquor (BL) from which a high volume of hydroxy acids (HAs) can be separated for further catalytic processing. Here, we explore the catalytic upgrading of HAs, including the conversion of (1) a model HA, gluconic acid; (2) a model mixture of HAs, and (3) a real mixture of HAs derived from kraft BL on M/Nb_2_O_5_ (M = Pd, Pt, Rh, and Ru). The hydrodeoxygenation of model gluconic acid reveals that “volatile” carboxylic acids (mainly C_2_ and C_3_), levulinic acid, and cyclic esters are significant products over all the catalysts, with Pd/Nb_2_O_5_ showing superior activity and selectivity toward valuable intermediates. The model mixture of HAs shows a wide range of reactivity over the supported metal catalyst, with the product selectivity strongly correlating to reaction temperature. Utilizing a 0.25% Pd/Nb_2_O_5_ catalyst, a real mixture of HAs derived from kraft BL is successfully dehydroxylated to produce a mixture rich in C_3_–C_8_ carboxylic acids that may be amenable for further upgrading, e.g., catalytically to ketones with high carbon chain lengths. Despite the feedstock complexity, we selectively cleaved the C–OH bonds of HAs, while successfully preserving most of the −COOH groups and minimizing C–C and C=O bond scission reactions under the operating conditions tested. The BL-derived HA stream is thus proposed to be a suitable platform for producing mixed carboxylic acid products from an overoxygenated byproduct feed.

## Abstract 2

This study employs a mixture of hydroxy acids derived from the kraft pulping of renewable circular resources (i.e., woody biomass) to produce platform chemicals suitable for upgrading to biochemical, toward contributing to sustainable development.

## Introduction

The production of chemicals and materials from renewable or circular resources is an important aspect of sustainable development.^1^ Utilization of biomass resources has been the subject of extensive research for sustainable production of commodity and specialty chemicals as well as liquid fuels.^2,3^ Lignocellulosic biomass (LCB) is the most abundant biomass resource,^4^ but it requires deconstruction (e.g., into various types of molecular feedstocks) before it can be upgraded by numerous proposed pathways.^5^ Because oxygen removal is a key step in the processing of biomass-derived feeds, many approaches involve supported metal catalysts under hydrogenating conditions.^6^ Over the years, researchers have designed and explored different catalytic systems to transform lignocellulose-derived carbohydrates including glucose to sorbitol,^7^ fructose to mannitol,^8^ furfural to furfuryl alcohol, furan, and tetrahydrofuran,^9,10^ etc. Further upgrading of these intermediate products (sorbitol, mannitol, furfuryl alcohol, etc.) has been explored, targeting different platform chemicals, transportation fuels, and other products.

**Figure 1:**
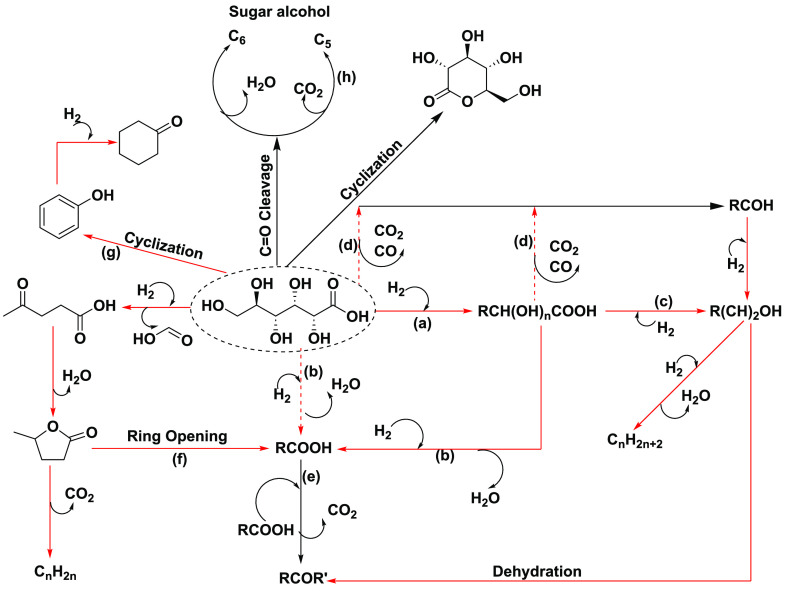
Expected reaction routes in hydroxy acids (HAs) upgrading.

Many intermediate products can be formed during the hydrodeoxygenation (HDO) of the biomass-derived carbohydrate substrates resulting from the hydrogenolysis of C–C, C–O, −O–C–O, and C=O bonds.^11^ For instance, Figure 1 depicts the expected reaction pathways during the HDO of an oxidation product of glucose (i.e., gluconic acid). In Figure 1, route (a) produces hydroxy acids with lower carbon atoms (such as lactic, glycolic, and malic acids), typically owing to incomplete C–OH removal. Meanwhile, in route (b), reaction conditions favor the complete cleavage of the C–OH bonds to form carboxylic acids of varying carbon numbers.^12^ Route (c) is the direct hydrogenation of the C=O carbonyl group of HAs to alcohols,^13,14^ whereas (d) represents C–C cleavage on metal sites forming aldehydes with the release of CO_2_ and/or CO via decarboxylation and decarbonylation, respectively.^11,15^ Route (e) is the ketonic decarboxylation of carboxylic acids to ketones,^16,17^ (f) is the ring opening reaction of γ-valerolactone (a cyclic ester) to form pentanoic acid on strong acid sites,^18^ and (g/h) represent a combination of hydrogenation, dehydration, and cyclization reactions, due to intramolecular interactions between hydroxyl and carbonyl groups.^19^ The complexity of these pathways, with different bond cleavages occurring, suggests the importance of developing a catalytic system capable of selectively cleaving a specific type of bond from the biomass-derived oxygenates toward achieving a target product.^20^

Transition metals (especially, Pd, Rh, Pt, and Ru) have been conventionally employed in the HDO of biomass oxygenates owing to their ability to activate hydrogen into a form that is suited for the C–O removal process.^21,22^ In many research studies, the roles of metal sites have been extensively studied using model and real carbohydrate feedstocks.^21,23,24^ For instance, during the HDO of sorbitol, Pd and Pt metal sites played the role of hydrogenating C–O and C=O bonds formed during the dehydration reaction. The metal sites additionally catalyzed the C–C bond cleavage reaction leading to decarboxylation/decarbonylation products.^20,25^ In another investigation that assessed the roles of metals (Ru, Pt) in glycerol hydrogenolysis, it was noted that the more a metal possesses available unoccupied orbitals, the more likely it is to drive C–C bond cleavage reactions.^26^ Despite the crucial roles of metal sites in HDO reactions, they sometimes do not offer satisfactory activity and selectivity to target products.^12^ Hence, noble metals are typically combined with acidic solid supports to form bifunctional metal–acid site pairs.^12^ With the presence of metal–support interactions, the metal site can enable the spillover of activated hydrogen atoms to the surface of the support, thereby facilitating the hydrogenation of chemical bonds.^26^ Thus, the metals perform the role of dissociating H_2_ and delivering the dissociated H atoms to the oxygenate, while the support provides the catalytic site for the surface reaction of the oxygenate and the H atoms.^19^ Consequently, the efficiency of the metal is improved. Toward elucidating the role of support sites, several candidates including activated carbon, ZrO_2_, TiO_2_, Nb_2_O_5_, etc. have been explored.^27−30^ In selecting the support materials, properties such as hydrothermal stability, the potential to sinter metal particles, the strength and density of acid sites, and other factors are important.^11,12^ Sintering of metal particles can depend on the nature of the metal oxide support. For instance, a study reported very quick and heterogeneous sintering of Pt particles on SiO_2_ and Al_2_O_3_ supports; however, these particles are stable on ZrO_2_.^31^ In another investigation, strongly acidic TiO_2_–WO_*x*_ supported on various metals (Ir, Pd, Pt) showed very good hydrothermal stability.^20^

The removal of the −OH functional groups in biomass oxygenates via C–O hydrogenolysis can be achieved either directly on metal sites or over bifunctional sites via stepwise dehydration and hydrogenation reactions.^32^ Meanwhile, in different systems involving the catalytic transformation of oxygenates, monometallic catalysts on carbon supports (e.g., Pt/C, Pd/C, etc.) have been observed to preferentially drive C–C cleavage reactions rather than C–O bond cleavage.^33^ Since the dehydration reaction step is catalyzed by the strong acid sites of the support, studies have explored the roles of many support materials. An example is the study of C–C and C–O hydrogenolysis in glycerol over Pd and Ru supported on ZrO_2_ and ZrO_2_/WO_3_.^20^ The electron density of the metals is reduced on ZrO_2_ compared to the ZrO_2_/WO_3_ dual support. The addition of WO_3_ on the ZrO_2_ support enhanced the electron density of the metals, thereby inhibiting the C–C cleavage reaction.^26^ This was attributed to the increase in the density of acid sites in the dual support relative to ZrO_2_ alone, thereby favoring the C–O bond cleavage reaction. Another study using Pt supported on Nb_2_O_5_ and Vulcan carbon also found the strong acidity of Nb_2_O_5_ in the upgrading of lactic acid to favor C–O bond cleavage, relative to C–C bond cleavage.^32^

Furthermore, depending on the oxygenate feed and target products, bimetallic catalysts can be superior to monometallic catalysts. The catalytic bifunctionality can be achieved either via metal/metal or metal/metal-oxide interactions.^34^ The addition of a second metal modifies the activity of the first metal by shifting its d-band density of states.^34^ For example, Dumesic et al. designed an approach to convert carbohydrates to hydrocarbons using a combination of different catalytic systems.^35^ A flow reactor packed with a Pt–Re/C catalyst drove the conversion of polyols and sorbitol to liquid organic products consisting of alcohols, ketones, carboxylic acids, and heterocyclic compounds. Ideally, C–C bond cleavage is more favored on Pt for oxygenated feedstocks than C–O bond cleavage. Meanwhile, a second metal (i.e., Re with oxophilic properties) modified the selectivity toward favoring C–O cleavage.^36^ However, most of the proposed mechanisms for HDO involves dual sites, with the oxide support providing the site for C–O activation. This can be achieved by metal/metal-oxide interaction, especially when the metal oxide has oxophilic properties (e.g., Fe_2_O_3_, Nb_2_O_5_, etc.).^28,37^

Black liquor (BL) is generated in large amounts (estimated >1 billion metric tons/year globally) in the pulp and paper industry utilizing the kraft process.^38,39^ In addition to lignin, inorganic sulfur salts, alkali, and carbonates, BL contains a range of C_1_–C_6_ acids.^40^ A typical kraft mill generating 4 MMT/yr of BL can produce about 0.1–0.2 MMT/yr of hydroxy acids (HAs). BL-derived HAs have 1–2 carboxylic (−COOH) groups and 0–5 hydroxy (−OH) groups.^41^ The total concentration of HAs in kraft BL is 3–5 wt %, comparable to the concentration of lignin and inorganics in BL. The carboxylic acids in BL contain volatile acids (formic acid and acetic acid) and nonvolatile compounds as well (hydroxy mono/dicarboxylic acids and dicarboxylic acid).^42^ In the context of this study, the entire carboxylic acid mixture is collectively referred to as “hydroxy acids” (HAs). During the delignification of wood in the kraft pulping process, the carbohydrates (i.e., mainly cellulose and hemicellulose) undergo a primary peeling reaction in alkaline pH at high temperatures (≥80 °C) to form different aliphatic carboxylic acids, with the elimination of the monosaccharide units.^42,43^ The peeling reaction competes directly with a stopping reaction that avoids the complete degradation of carbohydrates into carboxylic acids that are not conducive to an alkaline peeling reaction.^43^ Ideally, a greater extent of peeling of hemicellulose is expected relative to cellulose owing to its lower degree of polymerization as well as its amorphous structure.^43^ Since the content of cellulose and hemicellulose varies in different wood types, optimization of the kraft pulping operating conditions to modulate the composition of the hydroxy acids would be beneficial for catalytic conversion purposes. In current practice, the HAs are instead combusted along with lignin in the recovery boiler, thereby generating steam and electricity. Since the heating value of the carboxylic acid fraction is ∼50% less relative to lignin in BL,^44^ a significant effect of isolating the carboxylic acids on the energy supply of the paper mill is not expected.^43^ Thus, the recovery and subsequent valorization of the carboxylic acid stream to produce products with high market value is worthwhile. Recently, separation processes based on graphene oxide membranes and granulated activated carbon adsorbents have been developed for the scalable and continuous fractionation of BL into lignin-rich and HA-rich streams. In particular, HA streams with >90% acid purity are produced.^40,41,45^ This advance enables the use of the HA stream as a feedstock for producing value-added chemicals via a heterogeneous catalysis pathway.

One of the challenges for catalytic conversion is the complexity of the overoxygenated HA feedstock, exhibiting species with different structures, functionalities (C=O, −COOH, −OH), and overall composition. As shown in the expected reaction pathways in Figure 1, the production of carboxylic acids via route (b) is preferred, as its further upgrading via ketonization reactions to increase the carbon chain length is well-established in the literature.^17^ This requires the development of an appropriate catalytic dehydroxylation system to remove the −OH functional groups via selective C–O bond cleavage while preserving other functional groups (i.e., C=O and −COOH), and minimizing C–C bond cleavage reactions. To achieve this, a bifunctional catalyst comprising a metal site in synergy with a strong acid site is preferred. This is because the presence of strongly acidic support can improve the hydrogenation efficiency of metals, and the strong metal–oxygen bond formed from this synergy is required to drive the HDO of biomass oxygenates.^34^ Group VIIIB transition metals (e.g., Pt, Pd, Ru, and Rh) are known for their excellent ability, they favor C–C bond cleavage relative to C–O bond cleavage when supported on carbon.^36,46,47^ However, when used in tandem with reducible supports (Nb_2_O_5_, TiO_2_, CeO_2_, and ZrO_2_), C–C bond cleavage can be suppressed.^11,34^ The strong metal–support interaction created when metals are supported on reducible metal-oxides has been shown to correlate with excellent HDO activity relative to nonreducible supports like Al_2_O_3_, SiO_2_, etc.^48,49^ Particularly, Nb_2_O_5_ is an interesting support for our application owing to its hydrothermal stability,^28^ redox properties,^50^ its ability to promote C–O bond cleavage reactions,^51,52^ its oxophilicity,^53^ and its suitable acidity.^54,55^ In an investigation where a noble metal was supported on Al_2_O_3_, TiO_2_, ZrO_2_, and Nb_2_O_5_, the niobia provided the lowest C–O bond-breaking energy for phenol, suggesting its ability to strongly adsorb the C–O bond and reduce its dissociation energy, consequently promoting C–O bond cleavage.^56^ More so, the Nb^4+^/Nb^5+^ of Nb_2_O_5_ can act as an oxophilic site that can strongly adsorb the oxygen in the oxygenate substrate, thereby contributing to excellent adsorption of oxygenates on Nb_2_O_5_ and promoting the activation of C–O bonds.^28^ Hence, the presence of a strong acid support like Nb_2_O_5_ in the catalytic system is a viable strategy to efficiently supply H atoms for the target reaction.

Some of the acids making up the HAs (such as lactic acid and succinic acid) have been individually studied for catalytic conversion.^57,58^ For instance, lactic acid can react over Pt/Nb_2_O_5_ to form mainly acetaldehyde and propionic acid.^32^ To the best of our knowledge, no previous works have attempted to catalytically upgrade a complex mixture of model or realistic C_1_–C_6_ hydroxy acids sourced from the kraft pulping process. This paper presents the first known demonstration of the catalytic upgrading of model and realistic kraft black liquor-derived hydroxy acids on bifunctional catalysts. An overall objective for the catalytic upgrading of kraft BL-derived HAs involves the conversion of the enriched C_1_–C_6_ HA feedstock to C_15_–C_40_ range (partially or fully) deoxygenated mixtures. Here, we primarily focus on the first upgrading step, i.e., bifunctional catalysis to remove the −OH functionality via C–OH bond scission reactions. Catalytic experiments were systematically performed under different reaction conditions and feeds, and the results were analyzed in the context of understanding the substrate reactivity. First, gluconic acid (2,3,4,5,6-pentahydroxyhexanoic acid), a C_6_ monocarboxylic acid with 5 −OH groups, was used as a model substrate to understand the reactivity of highly oxygenated HAs. Then, a complex model mixture of eight HAs (Table S1 and Figure S1) with varying carbon number and OH content was studied. Finally, we studied the conversion of a real HA mixture separated from a kraft BL, containing 27 different acids (Table S2 and Figure S2). Overall, the HA feed is converted to a mixture of carboxylic acids, ketones, aromatics, esters, and hydrocarbons (removing up to 60% of the oxygen), along with some CO_2_ generation. We thus demonstrate that real HA feedstocks can serve as a platform to produce intermediates suitable for further catalytic upgrading into higher-value products, such as lubricants, waxes, and other materials.

## Materials and Methods

The complete details of experimental approaches are provided in the Supporting Information. This includes the materials and chemicals used, catalyst synthesis, catalytic reactor setup, preparation of HA feedstocks, reactor operation for evaluating catalyst performance, analytical methods, characterization of catalysts, and formulas for estimating catalyst performance. Relevant literature references are also cited therein.

## Results and Discussion

Due to the complexity of the HA mixture (Table S2) derived from the kraft process, initial catalytic experiments were conducted using model acids. The aliphatic carboxylic acids isolated from kraft BL are mainly composed of volatile acids (formic acid and acetic acid), hydroxy acids with 2–4 carbon atoms (mainly glycolic acid, lactic acid, 2-hydroxybutanoic acid, and 2,5-dihydroxypentanoic acid), and isosaccharinic acids with 5–6 carbon atoms (mainly gluco- and xylo-isosaccharinic acids).^41,59^ Some of these predominant carboxylic acids in kraft BL have been upgraded. For example, the utilization of the volatile components of the carboxylic acids (formic and acetic) via pathways like ketonization and catalytic transfer hydrogenation is well established in the literature.^60,61^ More so, studies on 2-hydroxypropanoic acid (i.e., lactic acid) via different catalytic systems to produce valuable platform chemicals have been reported.^62−65^ Particularly, the work of Dumesic and coworkers on lactic acid C–O hydrogenolysis to propanoic acid is insightful for understanding the behavior of similar hydroxy acids with higher carbon atoms.^32^ Hence, studying the reactivity of isosaccharinic acids via HDO pathways is crucial to have a complete understanding of the hydroxy acid mixture derived from kraft BL. Here, gluconic acid (a product of glucose oxidation) was selected as a model HA for individual study. This choice is justified because it is commercially available and possesses similar structural properties with the α- and β-saccharinic acid (i.e., gluco- and xylo-isosaccharinic acids) components of the BL which contribute ∼28 wt % of the realistic hydroxy acid mixture.

Gluconic acid possesses multiple oxygenated functional groups, low volatility, high water solubility, high reactivity, and low vapor pressure. We studied its conversion in the aqueous phase at suitable high pressure and mild temperatures.^32^ Details of the continuous flow reactor setup and the experimental procedures are presented in the Supporting Information. Qualitative and quantitative analyses of reaction products were achieved using gas chromatography–mass spectrometry (GC/MS) and a combination of gas chromatography and high-performance liquid chromatography (GC/HPLC), respectively, as illustrated in Tables S3–S5 and Figures S3–S12. We initially screened several group VIIIB metals as catalysts for the C–OH bond cleavage of the model gluconic acid molecule, specifically Pd, Ru, Rh, and Pt-supported on Nb_2_O_5_, due to their known hydrogenolysis activity and strong acidity, respectively.^11,66,67^ Pt/C was reported to be an efficient catalyst for C–C bond scission relative to C–O bond scission.^32,68^ However, the presence of acidic sites provided by Nb_2_O_5_ (i.e., Pt/Nb_2_O_5_ catalyst) favored the C–O bond cleavage via a 2-step reaction (dehydration–hydrogenation).^69^ Thus, we tested the HDO of gluconic acid with different nominal loadings of Pd (0.1–1.0 wt %) on the Nb_2_O_5_ support, and the results are shown in Table S6. The activity for gluconic acid HDO increased with increasing metal loading. At the lowest metal loading of 0.1 wt % Pd, about 25% of the converted carbon constitutes undesired hydroxy acids, which significantly decreased to ∼3% at 1 wt % Pd loading. The selectivity to desired target species such as carboxylic acids, levulinic acid, and esters is similar and more favorable at Pd loadings ≥0.25 wt %. Distinctively, an increasing selectivity to xylitol (a C_5_ sugar alcohol) was observed with increasing Pd loading (from ∼2% at 0.1 to ∼11% at 1.0 wt % loading), formed via C=O cleavage of the carbonyl group.^19^ Hence, in all the screening experiments, 0.25 wt % nominal metal (Pd, Ru, Rh, or Pt) loading was deemed sufficient to drive the hydrodeoxygenation (HDO) of gluconic acid. In preliminary experiments utilizing >20 wt % gluconic acid concentration, we observed reactor clogging leading to poor quantification of the product stream, as well as difficulty in dislodging the postreaction content of the reactor. Thus, a concentration of ∼11 wt % was selected to study the reactivity of gluconic acid during HDO reaction. The catalysts were operated at low reactant conversions (i.e., < 10%, differential reactor conditions) at 2.85 h^–1^ weight hourly space velocity (WHSV) of gluconic acid. The conversion and normalized product distribution of 11 wt % aqueous gluconic acid in the presence of H_2_ coflow are depicted in Figures S13 and S14. In the control experiment (quartz wool only, no catalyst), only 1% conversion was obtained (Figure S13). All four catalysts showed similar conversions of 6–8% and selectivities to the desired products such as carboxylic acids, esters, and ketones. The initial results suggested that all the catalysts can potentially drive HA conversion under these conditions, with the Pd and Pt catalysts indicating slightly higher activity and a more desirable product distribution with lower CO_*x*_ evolution.

**Figure 2:**
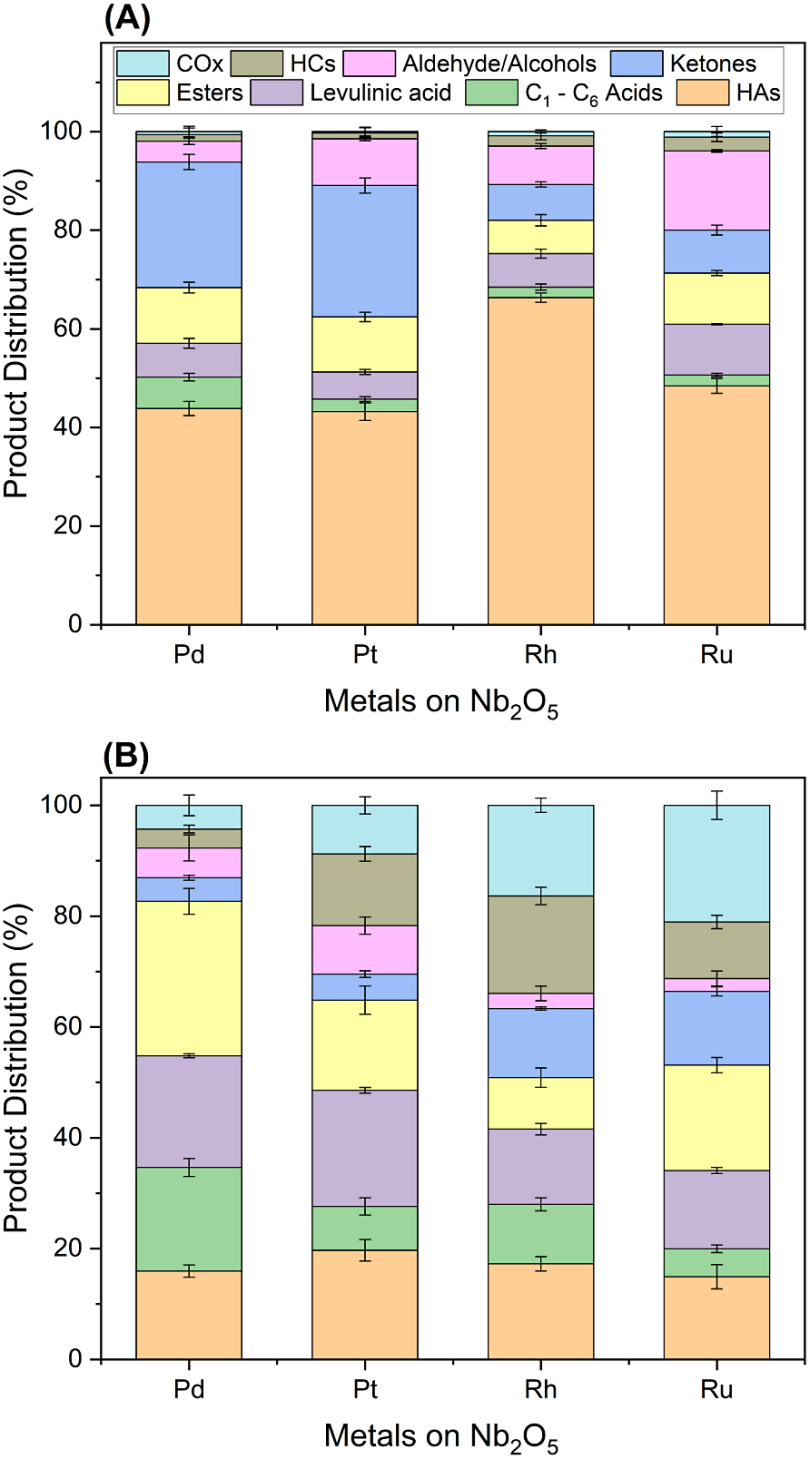
Product distribution for the hydrodeoxygenation of model gluconic acid on 0.25% M/Nb_2_O_5_ (M = Pd, Pt, Rh, and Ru) (A) without H_2_ coflow and (B) with H_2_ coflow. Operating conditions: 230 °C, 60 bar, 0.1 mL/min (equivalent to 2.85 h^–1^), 50 mL/min H_2_ coflow. Steady-state data were taken after 5 h on-stream. HAs represent organic acids containing −OH functional groups. HCs represent hydrocarbons. Carbon recovery: 86–89%.

Carbohydrate feedstocks such as glucose and polyols can typically undergo simultaneous deoxygenation and reforming reactions over bifunctional catalysts.^33^ During reforming, the carbohydrate-derived feedstock can adsorb on the metal sites and dehydrogenate via C–C cleavage to form H_2_ and CO_2_ via the water–gas shift reaction.^13^ The in situ-generated H_2_ can then drive the hydrogenolysis and hydrogenation reactions. Hence, we conducted gluconic acid conversions at a higher temperature (230 °C, 60 bar, and 2.85 h^–1^), with and without H_2_ coflow. Figure 2 depicts the product selectivity, and Figure S15 shows the conversions. Overall, the gluconic acid conversion is significantly higher with H_2_ coflow (79–94%) than without (50–68%). This suggests that in the case of in situ H_2_ generation, the amount of formic acid generated is insufficient to provide the H_2_ required to drive the conversion of gluconic acid comparable to that obtained with H_2_ coflow. Additionally, Figure 2 shows large differences in product selectivity between the two cases. Without H_2_ coflow, undesirable products containing −OH functionalities (arabinose, lactic acid, glutaric acid, glycolic acid, etc.) are formed significantly. The remaining (desirable) products are distributed between volatile carboxylic acids, ketones, esters, alcohols, aldehydes, and hydrocarbons. In contrast, H_2_ coflow shows a large increase in the selectivity to desirable products. The Pd-based catalyst showed the highest selectivity to C_1_–C_6_ carboxylic acids and esters, while the selectivity to levulinic acid is similar on both Pt and Pd catalysts. Among the ester products, up to 87% was contributed by γ-valerolactone (GVL), gluconic acid γ-lactone, and gluconic acid δ-lactone over the 0.25% Pd/Nb_2_O_5_ catalyst, which was the highest selectivity to cyclic esters among the current four catalysts. The data suggest that 0.25% Pd/Nb_2_O_5_ can efficiently cleave the C–OH bonds of gluconic acid to produce mainly levulinic acid (a keto acid), carboxylic acids, and cyclic ketones. The levulinic acid undergoes dehydration and hydrogenation on metal–acid sites to form GVL,^70^ while the gluconic acid lactones were formed via the cyclization of gluconic acid.^19^ We further compared the niobia-supported metal catalysts with carbon-supported metals at the same conditions (i.e., low conversion), and the results are shown in Figure S16. All catalysts supported on carbon revealed lower activity relative to metal/niobia catalysts. However, we observed a noticeable increase in selectivity to acids (including levulinic acid) and a decrease in the selectivity to esters, probably due to the absence of acid sites to drive dehydration reactions. Moreover, the M/carbon catalysts cleaved the C=O bond of gluconic acid to form C_5_ and C_6_ sugar alcohols (sorbitol and xylitol). All metal/carbon catalysts show some generation of CO_2_ (no CO was identified), which became more significant with an increased reaction temperature (Table S6). Gluconic acid can adsorb on the metal–acid sites and undergo decarboxylation and decarbonylation (C–C bond scission), dehydrogenation (C–H bond scission), or hydrogenation and dehydration (C–O bond scission) to form reactive intermediates (Figure 1). The selectivity toward CO_2_ and alkane/alkene hydrocarbons is highest over Rh and Ru catalysts, indicating that they increased the rate of C–C bond cleavage relative to C–O bond scission. Overall, the 0.25% Pd/Nb_2_O_5_ catalyst offered the most desirable conversion, carbon balance, and highest selectivity to intermediate oxygenates (e.g., esters, ketones, and C_1_–C_6_ carboxylic acids) that are good intermediates for the synthesis of value-added products.

Characterization techniques, including H_2_ chemisorption, ammonia temperature-programmed desorption (TPD), and N_2_ physisorption, were used to probe the physiochemical properties of the supported metal catalysts. Table 1 displays the total acid sites, metal dispersion, active particle diameter, and N_2_ physisorption parameters for all of the catalysts. Ammonia TPD profiles are shown in Figure S17. The supported metal catalysts exhibited lower total acidic sites (512–641 μmol/g) relative to the bare acidic niobia support (896 μmol/g) due to partial coverage of the Nb_2_O_5_ surface with the metal domains. The significant variation in the total acid sites in the supported metal (with the highest being 641 μmol/g for 0.25% Pd/Nb_2_O_5_) suggests that the metal domains exhibited different coordination with the surface of Nb_2_O_5_.^71^ The N_2_ physisorption isotherms and pore size distribution of Nb_2_O_5_ synthesized via the hydrothermal method (Supporting Information) are shown in Figures S18 and S19. All catalysts exhibit typical Type III isotherms with hysteresis in the adsorption–desorption curves, resulting in a mesopore distribution.^72^ Among the supported metal catalysts, 0.25% Pd/Nb_2_O_5_ revealed the highest BET surface area (141 m^2^/g) and pore volume (0.30 cm^3^/g). H_2_ pulse chemisorption was employed to elucidate the metal dispersion and the average particle diameter. As seen in Table 1, the metal dispersion follows the order Pd > Rh > Pt > Ru, with the 0.25% Pd/Nb_2_O_5_ showing ∼58% overall dispersion. This indicates that the Pd-based catalyst afforded the surface of acidic Nb_2_O_5_ with the highest fraction of exposed Pd metal atoms for C–OH bond scission. The catalytic performance exhibited by 0.25% Pd/Nb_2_O_5_ can thus be attributed to relatively more uniform Pd dispersion, high specific surface area, and high total acid sites, as confirmed by the characterization results. Hence, we selected a 0.25% Pd/Nb_2_O_5_ catalyst for subsequent experiments.

**Table 1:** Physiochemical Properties of the Supported Metal Catalysts Used in This Study^a^

catalysts	S/A^b^(m^2^/g)	PV^b^(cm^3^/g)	PR^b^(nm)	active d_p_^c^(nm)	dispersion^c^**(%)**	acid sites^d^(μmol/g)
0.25 Pd/Nb_2_O_5_	144	0.30	4.9	1.9	58	641
0.25 Pt/Nb_2_O_5_	132	0.25	5.0	4.3	31	632
0.25 Rh/Nb_2_O_5_	135	0.26	4.9	2.7	39	623
0.25 Ru/Nb_2_O_5_	137	0.25	5.1	14.1	10	512
Nb_2_O_5_	163	0.31	4.9	-	-	896
0.25 Pd/Nb_2_O_5_ (used)	94	0.19	5.2	-	-	-

a S/A = BET surface area, PV = pore volume, PR = pore radius.

b N_2_ physisorption.

c Chemisorption.

d Ammonia temperature-programmed desorption.

**Figure 3:**
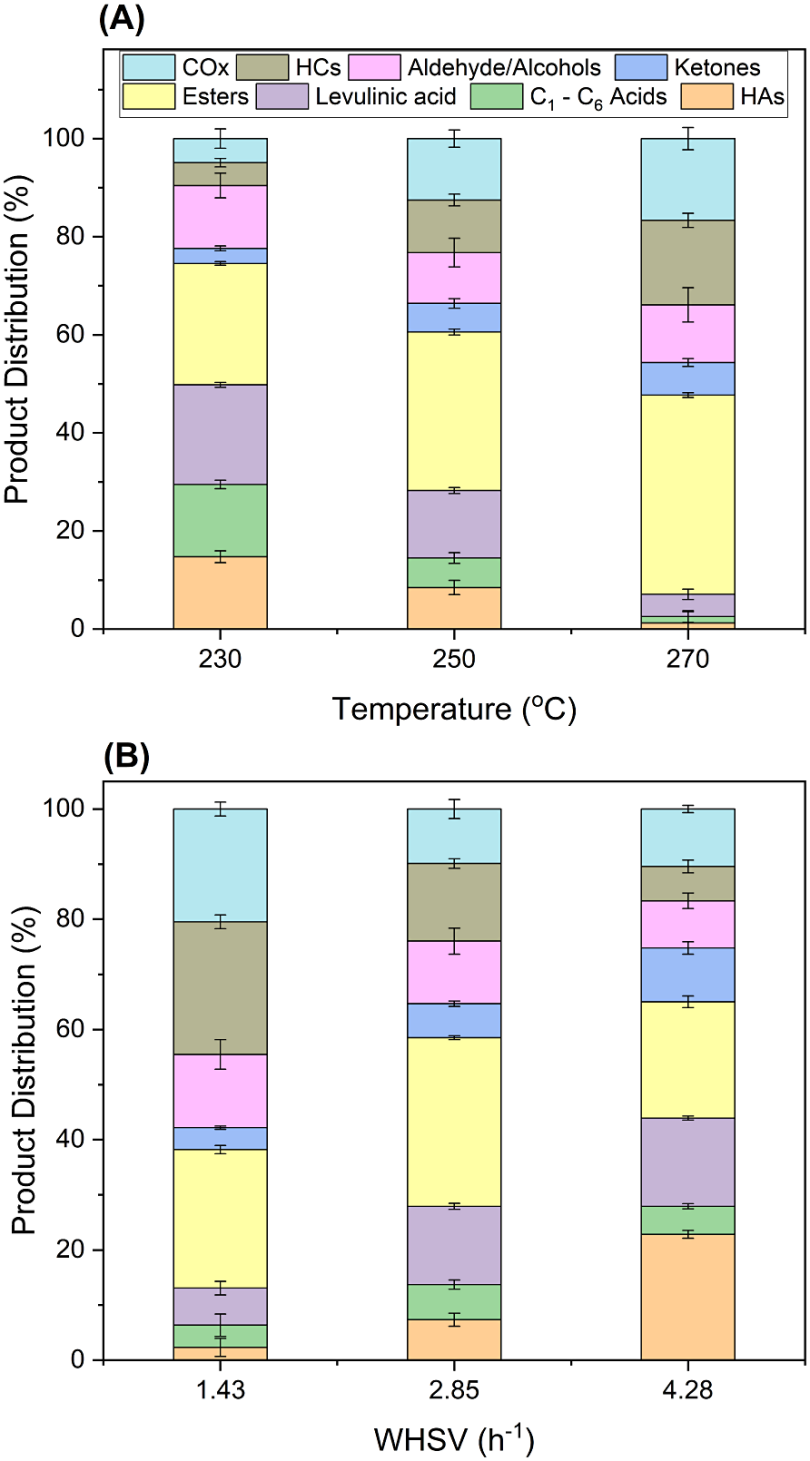
Product distribution for the hydrodeoxygenation of model gluconic acid on 0.25% Pd/Nb_2_O_5_ at (A) temperatures between 230 and 270 °C at 60 bar, 2.85 h^–1^, 50 mL/min H_2_ coflow and (B) weight hourly space velocity (WHSV) between 1.43 and 4.28 h^–1^ (equivalent to 0.05 to 0.16 mL/min) at 60 bar, 250 °C. Steady-state data were taken after 5 h on-stream. HAs represent organic acids containing −OH functional groups. Carbon recovery: 80–88%.

We next explored a wider range of operating conditions (temperature, WHSV, and pressure) to guide the subsequent processing of the more complex mixed HA feedstock. Gluconic acid is thermally unstable, and it is important to convert it at a sufficiently low temperature to minimize the contribution of degradation reactions. Generally, gluconic acid conversion increased with temperature (Figure S20), achieving close to 100% conversion at 250 °C. Figure 3 shows the effect of temperature on product distribution over 0.25 wt % Pd/Nb_2_O_5_ at a constant WHSV of 2.85 h^–1^ and 60 bar pressure. At the lowest temperature (230 °C), gluconic acid conversion was high (90%), but 11% of the detected carbon products constituted acids with −OH functional groups (i.e., HAs). At higher temperatures, the selectivity to HA products decreases (Figure 3a), suggesting favorable conversions of intermediate HAs such as glycolic and lactic acids. The selectivity to levulinic acid decreases with increasing temperature, indicating that levulinic acid is consumed via hydrogenation to GVL. The selectivity to carboxylic acids (which are ∼90% acetic and propionic acids) decreases with increasing temperature. The pathway to ketones (mainly C_6_ cycloketones) is favored at higher temperatures. The presence of Nb_2_O_5_ has previously been reported to catalyze the ketonization of propionic acid to 3-pentanone.^32^

Figure 3b shows the effect of WHSV at a fixed temperature of 250 °C. Longer contact time (1.43 h^–1^ WHSV) led to decreased selectivity to HAs and increased conversion of levulinic acid to GVL; however, the selectivity to esters decreased at the expense of hydrocarbon products (mainly 2-hexene). This could be attributed to the favorable decarboxylation of GVL under this condition to form butylene, which possibly undergoes conversion to 2-hexene. Furthermore, at the shortest contact time tested (4.28 h^–1^), the selectivity to HAs became significant. Figure S21 depicts the variation of the product distribution with pressure (35 and 60 bar). The only notable difference between the two pressures was the lower carbon recovery at 35 bar. At this condition, the pressure is insufficient to hold the gluconic acid in the aqueous phase, causing partial plugging of the reactor inlet. This is evinced by the observation of a brown-colored caky solid at the inlet resulting from gluconic acid crystallization. Based on the above results, we selected 250 °C, 60 bar, and 2.85 h^–1^ as a suitable baseline operating condition for subsequent experiments.

**Figure 4:**
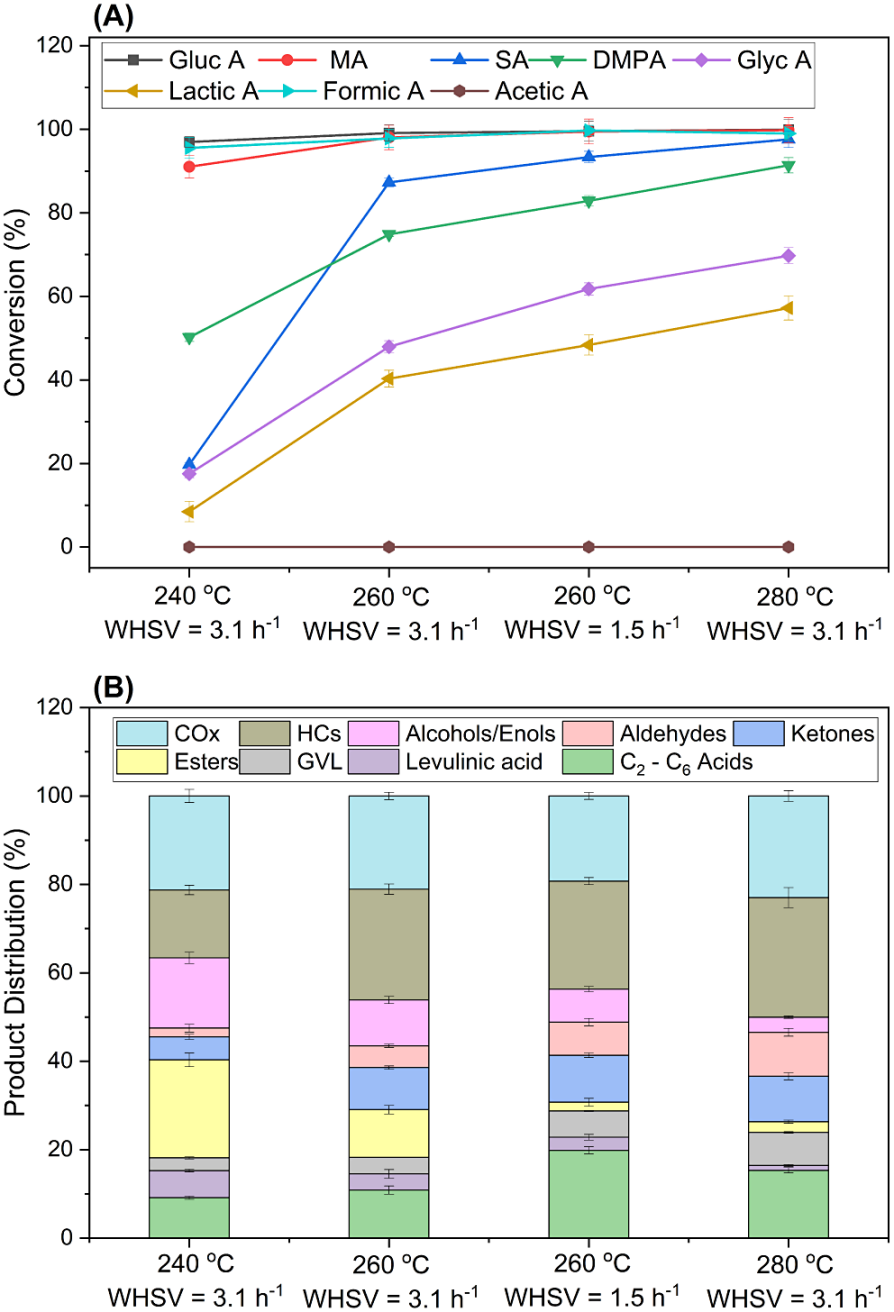
Catalytic performance of 0.25% Pd/Nb_2_O_5_ for the hydrodeoxygenation of model mixed hydroxy acids at temperatures between 240 and 280 °C. (A) Conversion of individual hydroxy acids. (B) Product distribution. The data for longer contact time (i.e., 1.5 h^–1^ WHSV) at 260 °C are shown. Operating conditions: 60 bar, 50 mL/min H_2_ coflow. Steady-state data were taken after 7 h on-stream. Carbon recovery: 78–82%. Gluc A = gluconic acid, MA = malic acid, SA = succinic acid, Glyc A = glycolic acid, and DMPA = dimethylolpropionic acid.

Eight representative organic acids were used to formulate a model mixture feed (Table S1 and Figure S1). This mixture approximates the complete set of organic acids separated from black liquor, as shown in Table S2 and Figure S2. These acids are divided into 9 classes based on the number of −COOH (1–2) and −OH (0–4) groups present. Figure 4 depicts the effect of temperature (240–280 °C) and WHSV at constant pressure (60 bar) and H_2_ flow (50 mL/min) on the conversion of each of the acids in the model mixture over 0.25% Pd/Nb_2_O_5_. Increased temperature increased the total conversion of HAs, suggesting faster reaction rates. The individual conversions of each acid also generally increased with temperature, but the acids showed a wide range of reactivity (Figure 4a). At 260 °C and WHSV of 3.1 h^–1^, ∼38% of lactic acid and ∼43% of glycolic acid were converted. However, using these two molecules individually as reactants at comparable reaction conditions resulted in conversions of ∼70% and ∼48%, respectively, and widely different product distributions (Figures S22 and S23). In Figure S23 (conversion of glycolic acid feed), ∼20% of the carbon selectivity is toward the lactic acid product. This indicates other parallel reactions during the conversion of the 8-component mixture, leading to the production of lactic acid and hence a lower conversion. Similarly, no acetic acid conversion is seen in Figure 4a, but a control experiment using 10 wt % acetic acid alone (Figure S24) at 280 °C/40 bar showed ∼6% conversion on 0.25% Pd/Nb_2_O_5_. This suggests that acetic acid is converted to some extent in the mixture feed, but acetic acid formation via C–OH bond cleavages in the other acids also occurs. Thus, some components in the HA mixture feed can also appear as products of reactions of larger HAs.

The product distributions are shown in Figure 4b, and Table S7 shows a detailed analysis of the product selectivity groups. In general, the combined selectivity to oxygenated products decreased from 62% at 240 °C to 50% at 280 °C, with the rest of the carbon detected in the product mixture being CO_2_ and hydrocarbons. At a constant WHSV of 3.1 h^–1^, higher temperature favored selectivity to carboxylic acids (C_2_–C_6_), from ∼9% at 240 °C to 15% at 280 °C, reaching 19% at longer contact times (1.5 h^–1^). Levulinic acid selectivity declined (and GVL selectivity increased) with increased temperature, suggesting that more levulinic acid was consumed on the catalyst sites to form GVL. As shown in Table S7, aromatics, anhydrides, and esters were observed to dominate at the lowest temperature, and their fraction decreased at higher temperatures. Two new species (isobutyraldehyde and isobutanol) were detected, probably due to the addition of C_4_–C_5_ HAs in the model mixed HA feed. These products were not detected during the conversion of gluconic acid, lactic acid, or glycolic acid alone. The selectivity for aldehydes (acetaldehyde and isobutyraldehyde) and alcohols increased with temperature. Aldehyde formation suggests decarboxylation via C–C bond cleavage on the Pd site with CO_2_ release.^32,68^ The aldehydes can form alcohols by direct hydrogenation, i.e., isobutyraldehyde to isobutanol and acetaldehyde to ethanol (Figure 1). There is significant selectivity for CO_*x*_ (CO_2_ and CO) that increases with temperature (Figure 4b). This can mainly be attributed to the decomposition of formic acid into H_2_ and CO_2_. Alternative pathways to CO_2_ during the hydrodeoxygenation process are also shown in Figure 1. The comparison of data from Pd/C and Pd/Nb_2_O_5_ in the HDO of model mixed HAs revealed that Pd/C is less active (47% versus 72% conversion) and generated significantly more CO_2_ than Pd/Nb_2_O_5_ at the same operating conditions (Table S7). Meanwhile, 0.25 Pd/C offered a comparably superior selectivity to alcohols, levulinic acid, and aldehydes, probably due to the absence of catalytic sites to drive their further conversion during the HDO reactions. Finally, hydrocarbon products including light alkanes, light olefins, and 1-hexene/2-hexene were detected, with higher temperatures leading to higher hydrocarbon selectivity.

The organic acids making up the model mixture of HAs showed a wide range of reactivities on the niobia-supported Pd catalyst. Also, a strong correlation between reaction temperatures and product selectivity was observed. While low temperatures revealed selectivity to enols and esters, higher temperatures are selective to carboxylic acids, ketones, and aldehydes. Based on the finding that the presence of formic acid in the model HA mixture primarily determines the CO_2_/CO levels in the product stream, a good strategy to either utilize formic acid or control its production during the peeling reaction of carbohydrates in wood pulping is important. Overall, the partial HDO of mixed model acids revealed the importance of identifying a suitable combination of operating conditions (reaction temperature, pressure, WHSV, and H_2_ flow rate) to achieve an appropriate degree of hydrodeoxygenation and a more desirable product distribution for further upgrading.

The operating conditions determined from the conversion of the model acid feed were used as a starting point for HDO of the real HA mixture feed derived from kraft BL. This mixture (80 wt % acids) was diluted to ∼8 wt % before use. The stability of the kraft BL-derived HAs was evaluated by catalyst-free control experiments conducted at 280 °C (the temperature used for converting the model acid feed) and at 330 °C. As shown in Figure S25, the feedstock becomes increasingly unstable as the temperature increases, with ∼25% conversion at 280 °C and ∼38% conversion at 330 °C. Carbon recovery decreased at the highest temperature tested. Gluco-isosaccharinic acid (GISA, ∼25% in the HA feed) appeared the most sensitive to temperature. At 330 °C, the oxygenates formed from the converted carbon species include volatile acids (mainly methacrylic acid), ketones (mainly acetone), acetaldehydes, alcohols, and esters. Based on these findings, as well as the optimum operating conditions in the HDO of mixed model HAs, a temperature range of 265–295 °C was chosen for further exploration.

**Figure 5:**
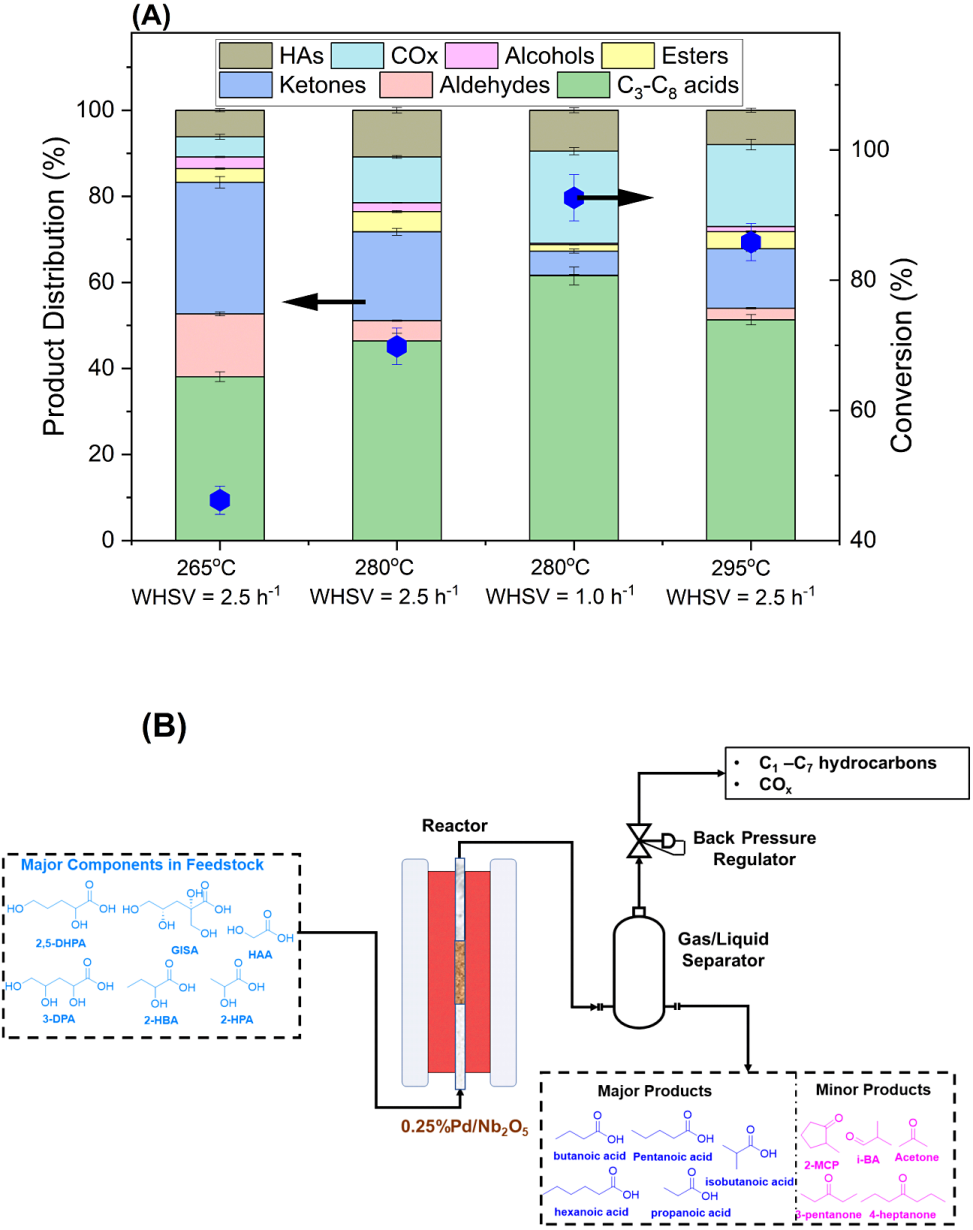
Catalytic performance of 0.25% Pd/Nb_2_O_5_ for the hydrodeoxygenation of kraft BL-derived hydroxy acids. (A) Total HA conversion and product distribution at temperatures between 265 and 295 °C. The data for longer contact time (i.e., 1.0 h^–1^ WHSV) at 280 °C are additionally shown. (B) Reactor configuration for the catalytic conversion of BL-derived HAs reported in this study, showing the major reactants and products. Operating conditions: 60 bar, 50 mL/min H_2_ coflow. The data were taken after 7 h on-stream. Carbon recovery: 66%–71%.

Figure 5 summarizes the HDO of ∼8 wt % aqueous HAs over 0.25% Pd/Nb_2_O_5_ at different temperatures (265, 280, and 295 °C), WHSVs (2.5 h^–1^ and 1.0 h^–1^), and at 60 bar pressure along with 50 mL/min H_2_ coflow. In the HDO step, the experiments were designed to completely remove the −OH functionality from the HA feedstock and produce a stream rich in a particular class of products (e.g., carboxylic acids, esters, or ketones). These are desirable for subsequent conversions to targeted chemical products, which will be the subject of future work. Due to favorable kinetics, the total conversion of HAs increases with temperature (Figure 5a) at WHSV = 2.5 h^–1^. Many of the acids comprising the BL-derived HA feedstock are temperature-sensitive, and their OH groups can be easily removed in the presence of 0.25% Pd/Nb_2_O_5_. However, glycolic acid, lactic acid, and 2,5-dihydroxypentanoic acid require further tuning of the operating conditions to achieve complete dehydroxylation. The total molar conversion of HAs increased from 45% at 265 °C to 86% at 295 °C, indicating incomplete dehydroxylation. This can be attributed to the formation of 2-hydroxypentanoic acid as the HDO reaction proceeds. We hypothesize that this intermediate HA was formed from the partial C–OH cleavage of GISA. The latter is very temperature-sensitive, and we observe a good correlation between its conversion and the formation of 2-hydroxypentanoic acid under all the conditions (including the control experiments). To achieve complete dehydroxylation, we increased the residence time by 60% (by loading more catalysts into the reactor and adjusting the H_2_/HA flow ratio) to obtain a lower WHSV of ∼1.0 h^–1^. At 280 °C and 60 bar, H_2_ coflow of 45 mL/min, and WHSV of 1.0 h^–1^, complete cleavage of the alcohol C–OH bonds was achieved, corresponding to a total conversion of ∼93% with the remaining 7% being acetic acid (Figure 5a). At these conditions, a total of ∼60% HDO of the feed stream was achieved, considering all oxygen present in the HA feed (Figure S26). Figure 5a additionally depicts the distribution of liquid and vapor products during HDO of HAs at varying operating conditions. Further details of the normalized aqueous product distribution are shown in Figure S27. Generally, the selectivity to carboxylic acids (C_3_–C_8_) increases with temperature, while the selectivity toward ketones decreases. This is contrary to the trend in ketonic decarboxylation, which is favored at higher temperatures.^73^ This finding suggests that the main ketones found in the product are not necessarily the primary products of ketonization. The selectivity to aldehydes decreases with increasing temperature, at the expense of alcohols. Correspondingly, the selectivities toward CO_2_ and hydrocarbons (C_1_–C_7_) increase with temperature.

The detailed product composition is listed in Table 2, corresponding to Figure 5a. At the most suitable reaction conditions (280 °C, 1.0 h^–1^), about 61% of the converted carbon is directed to C_3_–C_8_ carboxylic acid products, with propionic, *n*-butyric, *i*-butyric, and pentanoic acids comprising nearly all of this product class with very small amounts of hexanoic and 4-methyloctanoic acids. The high selectivity toward carboxylic acids is evident from the structural nature of the HAs in the feedstock, with >90% of the acids possessing an −OH functional group in their backbones, as well as the effectiveness of 0.25 wt % Pd/Nb_2_O_5_ in the selective cleavage of the C–OH bonds to form carboxylic acids. At reaction conditions unsuited for the complete dehydroxylation, increasing conversion of lactic and 2-dihydropentanoic acids led to a gradual increase in the selectivity to C_3_ and C_5_ carboxylic acids, indicating a correlation between the extent of conversion of these two HAs and the formation of C_3_/C_5_ carboxylic acids. Both HAs, though already present in the feed, are also generated during the reaction. At suitable conditions, there is 6% selectivity toward several ketones. The 3-pentanone and 4-heptanone products are attributed to the primary ketonization products of C_3_ and C_4_ carboxylic acids on Nb_2_O_5_. Alternatively, 1-butanol can undergo ketonization to 4-heptanone.^17,74^ The formation of 2-butanone can be attributed to the decarboxylation of a keto acid (i.e., levulinic acid).^75,76^ Similarly, acetone could have formed via the decarboxylation of 3-oxobutanoic acid. However, this species was not observed in the GC/MS qualitative analysis, likely due to its instability. The aldehydes (1% of the product carbon) formed via decarboxylation reactions during C–C bond scission of HAscan undergo hydrogenation to alcohols. Alternatively, alcohols can be formed via direct hydrogenation of HAs, as is evident from the formation of 1-pentanol.

**Table 2:** Detailed Product Selectivities in the Conversion of Kraft BL-Derived HAs Over 0.25% Pd/Nb_2_O_5_ at 280 °C, 60 Bar, 1.0 h^–1^, 50 mL/min H_2_ Coflow, Corresponding to Figure 5A

group	components	carbon	mol %	group	components	carbon	mol %
**Carboxylic acids**	acetic	2	0.77	**Ester**	γ-valerolactone	5	1.01
	propionic	3	25.10	**Alcohols**	methanol	5	0.23
	*n*-butyric	4	3.85		ethanol	2	0.09
	isobutyric	4	17.42		isobutanol	4	0.16
	2-methylbutyric	4	1.64		1-butanol	4	0.19
	methacrylic	4	0.01		1-pentanol	5	0.15
	pentanoic	5	11.17	CO***_x_***	CO_2_	1	17.42
	hexanoic	6	0.63		CO	1	0.15
	4-methyloctanoic	8	0.64	**Hydrocarbons**	methane	1	0.45
					ethylene	2	0.92
**Ketones**	acetone	3	2.33		1-propylene	3	2.34
	hydroxyacetone	3	0.01		1-butene	4	1.33
	2-butanone	4	0.77		pentane	5	0.37
	methylcyclopentenolone	6	0.18		1-pentene	5	0.01
	3-pentanone	5	0.99		2-hexene	6	4.19
	4-heptanone	7	1.15		hexane	6	0.76
	cyclohexanone	6	0.12		heptane	7	0.02
	2-methylcyclopentanone	6	0.13		cyclohexane	6	0.02
	3-methylcyclopentanone	6	0.11		methylcyclohexane	7	0.21
**Aldehydes**	acetaldehyde	2	0.18		benzene	6	0.44
	isobutyraldehyde	4	0.10		*m*-xylene	6	0.17
**Keto acid**	4-oxopentanoic acid	5	0.73		*o*-xylene	6	0.32
					ethylbenzene	8	1.25
					**Total**		**100**

The steady-state vapor product analysis revealed that ∼13% of converted carbon was directed to C_1_ −C_7_ alkanes (2%), alkenes (8%), and aromatic (2%) hydrocarbons. It is noteworthy that the 2-hexene product was observed in the aqueous product stream, while all other reported products under the hydrocarbon group are in the vapor product. The formation of C_2_–C_6_ olefins can be attributed to different sources, including dehydration of alcohol over Nb_2_O_5_ acid sites,^33^ oligomerization reactions of C_2_ and C_4_ light olefins,^77^ or dimerization of propylene.^78^ In a prior study, decarboxylation of γ-valerolactone over niobia-containing catalysts released butylene as the main product.^35^ In a different investigation, the dimerization of propylene to hexene occurred at sufficiently high reaction temperatures (250 °C).^78^ Alkane formation can be attributed to the dehydration of alcohols followed by subsequent hydrogenation, while aromatic hydrocarbons from biomass feedstock have been previously attributed to stepwise cyclization, dehydrogenation, and aromatization reaction steps (Figure 1).^79^ The selectivity to CO_*x*_ reached ∼18%, which is nearly all CO_2_ with a small amount of CO. Given this composition, separation of the hydrocarbons and other easily condensable gas/vapor products would allow for capture of a high-purity CO_2_ stream for storage or utilization.^80,81^ Finally, Figure 5b shows a schematic summarizing the main product streams from the catalytic HDO of the BL-derived HA feed.

**Figure 6:**
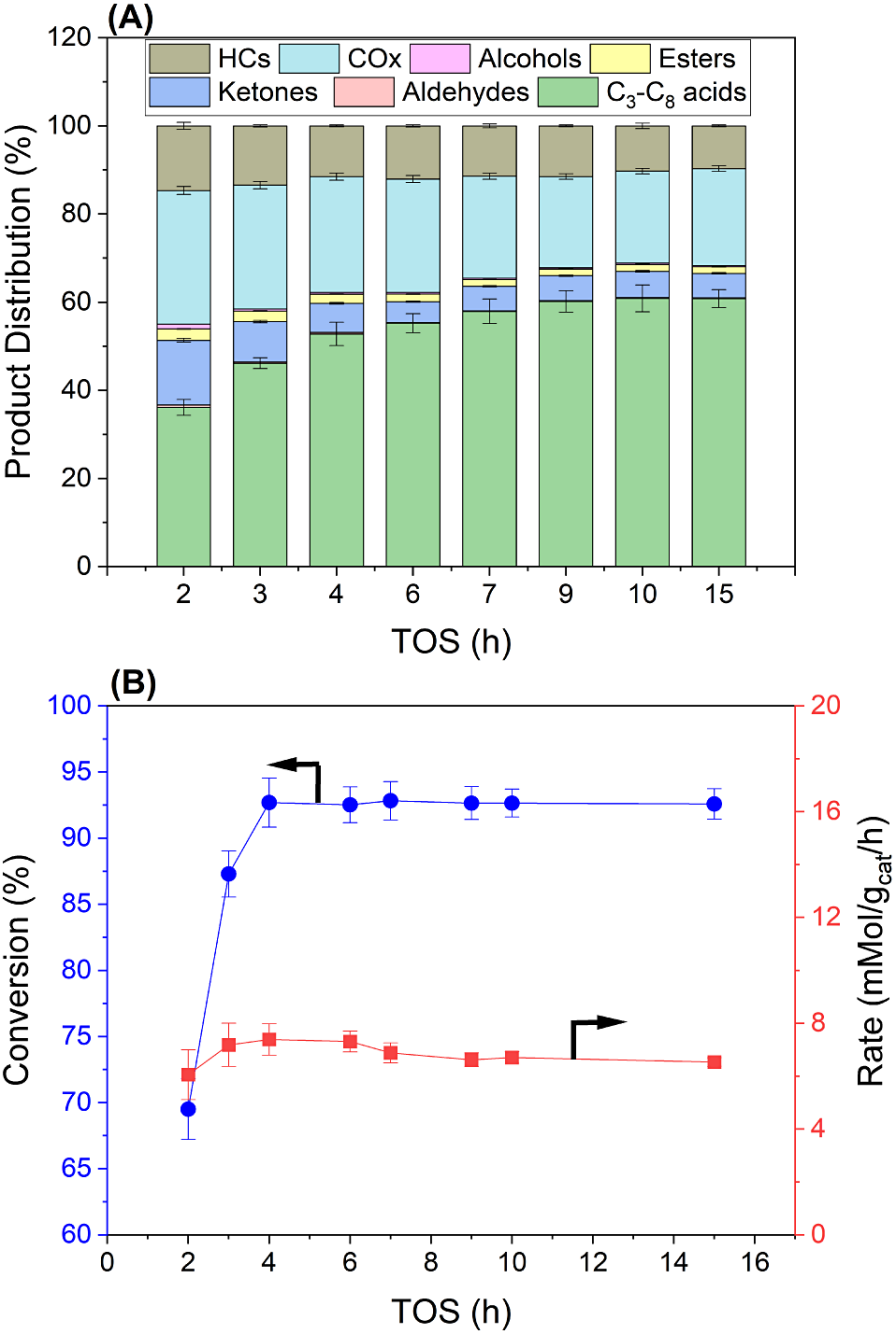
Catalytic performance of 0.25% Pd/Nb_2_O_5_ for the hydrodeoxygenation of kraft BL-derived hydroxy acids. (A) The product distribution as a function of time-on-stream (TOS) at optimum reactor conditions (280 °C, 60 bar, 1.0 h^–1^, 50 mL/min). (B) The conversion and hydrodeoxygenation rate as a function of time-on-stream at optimum reactor conditions. Carbon recovery: 66–71%.

The steady-state data reported in Figure 5a are taken at 7 h time-on-stream (TOS). To gain further insight into catalyst stability, extended conversion runs were performed at the suitable reaction conditions reported above. The transient data collected over 15 h TOS are depicted in Figure 6a,b. The normalized product distribution showed a steady increase in selectivity to C_3_–C_8_ acids in the first 4 h of the reaction, which was stabilized after 9 h. These TOS data suggest that the 0.25% Pd/Nb_2_O_5_ catalyst is stable under the current operation conditions. The powder XRD patterns of fresh and used catalyst samples are depicted in Figure S28, showing only a slight increase in pseudohexagonal Nb_2_O_5_ crystallite domain size from 12.2 nm (fresh) to 13.6 nm (used) based on the Scherrer equation. However, ∼34% decrease in the BET surface area was observed by N_2_ physisorption analysis (Figure S29 and Table 1).

The results from this study suggest that the mixture of hydroxy acids derived from kraft black liquor is a suitable mixture that may enable pathways to produce chemicals with a high market value. Figure S30 shows a diagram depicting the desired utilization of oxygenated products derived from the HDO reactions in this study. Upgrading the hydroxy acid stream to longer (C_20_ and higher) molecules could lead to higher-value products (e.g., biolubricants) that generate considerably more revenue compared to the current use of the hydroxy acids for combustion along with lignin to generate steam and electricity. As seen from our data, not all the carbon in the feed streams is recovered in the product streams (liquid and vapor). Although incomplete carbon balances are not uncommon in similar studies,^82^ it is worth reflecting on the likely causes of this mass balance nonclosure. These include loss of volatile products in the workup and transfer of samples after product collection, the presence of undetected light products, or the formation of heavy organic products that are trapped on the reactor walls, tubing, fittings, or catalyst surfaces. A potential solution in future studies could be to run the real HA feedstock with much higher concentrations (i.e., without dilution) to allow for substantial collection and analysis of heavy products. Furthermore, the pathways to the formation of hydroxy acids in BL are important in the context of catalytic conversion. While the content of cellulose in softwoods and hardwoods is similar, the hemicellulose in hardwood is typically greater,^43^ indicating that more hydroxy acids can be derived from hardwood than softwood. Thus, the hexoses, pentoses, and polysaccharides that determine the concentration of the hydroxy acids will vary with both wood type and cellulose/hemicellulose contents. Therefore, the HDO catalytic system must be designed to handle such variability.

In the context of catalytic upgrading of the mixture of HAs derived from kraft BL, the presence of formic and acetic acids is challenging. Formic acid is liberated during the peeling reaction, while acetic acid is formed via the hydrolysis of acetate groups of hemicellulose chains. The formation of these volatile acids is difficult to control during wood delignification, yet they are not particularly useful acids in complex, realistic HA mixtures from the perspective of efficient carbon utilization. This is especially so for formic acid, which gives off its carbon as CO_2_ during the HDO reactions. Thus, since both acids are commercially important intermediate chemicals, it may be more economically viable to isolate them from the HA mixture before catalytic processing. This way, the production of CO_2_ can be significantly minimized. Alternatively, the formic acid can be utilized via catalytic transfer hydrogenation as the source of hydrogen needed for the HDO reaction, without the need for an external supply of H_2_, but this would require tuning of the catalytic system. As shown in Table S8, although no significant Pd content was observed in the postreaction mixture that was accumulated for 10 h, there was evidence of loss from the solid catalyst after the reaction. Therefore, it is important to consider the mechanism(s) of leaching and strategies to minimize it.

## Conclusion

The hydrodeoxygenation of a kraft BL-derived HA feedstock was demonstrated, specifically targeting the removal of −OH functional groups via C–OH bond scission on acid-assisted metal sites (bifunctional sites). The reactivity of gluconic acid, afforded by its carbon and OH numbers, made it a suitable model candidate to initially study the HDO behavior of HAs on supported metal catalysts. Initial catalyst screening revealed that Pd, Pt, Rh, and Ru supported on Nb_2_O_5_ are potentially effective for the HDO process; however, the 0.25% Pd/Nb_2_O_5_ catalyst showed superior performance in the extent of deoxygenation and selectivity to carboxylic acids, levulinic acid, and cyclic esters (GVL and gluconic acid lactones). The use of mixed model acids to approximate a real kraft BL-derived HA feed proved beneficial in understanding this complex/multicomponent feedstock, where the concentration of formic acid in the feed was found to be the most significant contributor to the formation of CO_2_. In the final stage of this study, the kraft BL-derived HA feedstock containing at least 27 distinct HAs was successfully converted by HDO. Our catalytic studies revealed that 100% removal of OH functional groups can be achieved at 280 °C, 60 bar, 1.0 h^–1^, and 50 mL/min H_2_ coflow. These conditions selectively produced a C_3_–C_8_ carboxylic-acid-rich product stream, showing that the HA mixture is a practical feedstock for producing a carboxylic-acid-rich product stream amenable to the synthesis of high-value chemicals and materials in subsequent reaction steps. Finally, the catalytic performance as a function of TOS showed the Pd/Nb_2_O_5_ catalyst with a nominal metal loading of 0.25 wt % gave good stability over 15 h of operation, without sacrificing the selectivity to desired products. In doing so, this work also demonstrates the crucial role of establishing optimum reaction conditions to drive catalytic HDO of complex mixtures such as HAs on bifunctional active sites.

## Supporting Information Available

Detailed description of materials, methods, and mathematical formulas; Tables S1–S8 showing detailed compositions of mixed and realistic HA feedstocks; and Figures S1–S30 depicting the structures of HA feedstocks, GC/MS spectra, GC/HPLC peaks, catalyst performance, and characterization results (PDF)
