# Supplementary material for: Catalytic Upgrading of a Mixed Hydroxy Acid Feedstock Derived from Kraft Black Liquor
Source: ACS Sustain Chem Eng. 2024 Jun 3;12(24):9054–66. doi: 10.1021/acssuschemeng.4c00212 (PMC11191363; doi:10.1021/acssuschemeng.4c00212)
Supplement: Supplementary file 1 — sc4c00212_si_001.pdf [file sc4c00212_si_001.pdf]

Supporting Information

for

Catalytic Upgrading of a Mixed Hydroxy Acid Feedstock Derived from Kraft Black Liquor

Opeyemi A. Ojelade,[1] Qiang Fu,[1] Sankar Nair,[1*] and Christopher W. Jones[1*]

[1]School of Chemical & Biomolecular Engineering, Georgia Institute of Technology, Atlanta, GA 30332-0100, USA

[*]**Corresponding Authors:** cjones@chbe.gatech.edu; sankar.nair@chbe.gatech.edu;

Number of pages: 38

Number of supporting tables: 8

Number of supporting figures: 30

I. Experimental Methods

Materials and Chemicals

The kraft black liquor (BL) used in this work was obtained through a previously reported method.[17-18] All chemicals including H_2O_2 (30 wt%), ammonium niobate (V) oxalate hydrate (99.9 %), palladium(II) nitrate hydrate *t*etraammineplatinum(II) chloride hydrate, rhodium(III) nitrate hydrate, and ruthenium(III) nitrosyl nitrate, formic acid (FA), acetic acid (AA), lactic acid (LA, 90 wt%), glycolic acid (GA, 70 wt%), gluconic acid (50 wt%), succinic acid (SA), malic acid (MA), 2,2-bis(hydroxymethyl)propionic acid (DMPA), arabinose, glutaric acid, propionic acid, n-butyric acid, i-butyric acid, pentanoic aciud, hexanoic acid, 4 methyl octanoic acid, acetone, 3 pentanone, 4 heptanone, 2-butanone, cyclohexanone, methyl cyclopentanone, hydroxyacetone, acetaldehyde, isobutyraldehyde, levulinic acid, gamma-valerolactone, methanol, ethanol, isobutanol, 1-butanol, 1 pentanol, 2-hexene, were purchased from Sigma Aldrich (others: purity level $\geq$ 98 %). All chemicals were used as received except for those stated otherwise.

Catalyst Synthesis

Synthesis of Niobium Pentoxide (Nb_2O_5): The Nb_2O_5 support was synthesized via hydrothermal method, using ammonium niobate (V) oxalate hydrate (ANO) as the precursors. In a typical synthesis, 2.7 g ANO was dissolved in 33 mL H_2O and mechanically stirred for 5 minutes. This was followed by adding 7 mL of H_2O_2, and the mixture was transferred into a 60 mL Teflon-lined autoclave made of stainless steel (Parr). After sealing the reactor, the synthesis was carried out at 175 ^oC for 24 h followed by natural cooling. After cooling, the solid was separated from the solution using a centrifuge (ThermoFisher) washed several times with deionized water, and dried for 16 h at 80 ^oC. The obtained dried solid was ground using a mortar and pestle and calcined at

350 ^oC (ramp rate 2 ^oC/min, soak 3 h). The surface area was estimated as 144 m^2/g (Table 1 in the main text). The supported synthesized by this method is denoted by HT-Nb_2O_5-175.

Synthesis of TM/Nb_2O_5: Supported metal catalysts (TM = Pd, Pt, Rh, and Ru) were prepared using the impregnation method. Appropriate amounts of palladium(II) nitrate hydrate, Tetraammineplatinum(II) chloride hydrate, Rhodium(III) nitrate hydrate, and ruthenium(III) nitrosyl nitrate solution (Sigma Aldrich) precursors were used to achieving a target metal weight loading of 0.25% on Nb_2O_5 or carbon black supports. In a typical synthesis of 0.25Pd/Nb_2O_5, 0.0066 g Pd salt was allowed to completely dissolve in 1.4 mL H_2O, followed by a slow addition of 0.985 g HT-Nb_2O_5-175 for 15 minutes, and by vigorous stirring for 5 minutes. The obtained catalysts (slurry) were dried at 80 ^oC for 16 h and calcined at a temperature range of 400 ^oC (2 ^oC/min ramp) for 3 h to form Pd/HT-Nb_2O_5-175 with nominal Pd loading of 0.25 wt%. For the carbon support, dispersion in warm deionized water (~ 60 ^oC) was employed before the impregnation of metal solutions.

Catalytic Reactor

Liquid-phase hydrodeoxygenation reactions were performed in an up-flow, fixed-bed stainless steel reactor containing 3 heated zones heated externally by an adequately insulated furnace (A Testing System), and the reaction temperature was monitored by an Omega-type temperature controller/thermocouple. The H_2 gas flow was controlled by a Brooks-instrument type mass flow controller while the liquid (HAs in aqueous solution) was introduced into the reactor by an Isco pump (Teledyne 500D) via a 1/8″ Swagelok tubing. Both were directed to co-currently flow upward through the catalyst bed. The product stream from the reactor outlet flowed through a high-pressure gas-liquid separator. It was condensed at atmospheric temperature and collected periodically for HPLC (Supplementary HPLC analysis I) and GC (Supplementary GC analysis I) analyses. The gas separated from the liquid flowed through the back-pressure regulator (EquiliBAR ZF Series) which maintains the system pressure. Steady-state vapor samples flowing through the back-pressure regulator (BPR) were periodically collected into a sealed gas bag (Sigma-Aldrich) for offline analysis (see details below). For all experiments, the catalyst was packed in the middle of the reactor, and the remainder space was packed with quartz wool.

Feed Preparation

For experiments with gluconic acid, an 11 wt% aqueous feed was prepared by diluting commercially available 50 wt% gluconic acid (Sigma-Aldrich). The model mixed hydroxy acid feed was prepared by combining predetermined masses of formic acid, acetic acid, succinic acid, malic acid, lactic acid, glycolic acid, dimethylol propionic acid, and gluconic acid (all Sigma Aldrich) along with deionized (DI) water to achieve a total 20 wt% acid concentration in aqueous solution (**Table S1**). The real hydroxy acid (HA) product was separated from kraft BL[17-18] and contained 80 wt% acids. It was diluted to 8 wt% before conversion experiments. The detailed composition (31 individual acids) was determined by GC-MS analysis after derivatization, followed by GC quantification (as described in our previous work),[17] and the results are shown in **Table S2**.

Reactor Operation

After loading the catalyst, the temperature was raised to 300 °C (3 ^{o}C ramp rate) under H_2 flow (30 mL/min) and maintained for 2 h for reduction under 60 mL/min H_2 environment. After reduction, the reactor was cooled to the target reaction temperature and pressurized to the reaction pressure under H_2 flow. A reference pressure on the BPR was set through an N_2-controlled flow and assumed the reactor pressure. The reference inert gas pressure was supplied to the BPR at a pressure 5 bar higher than the pressure supplied by the ISCO pump. When the reaction pressure is reached (observed by exiting H_2 flow outside the BPR), the H_2 flow rate is set to the actual flow rate for the reaction. The HA feed flow was initiated (first 0.5 mL/min) to fill the tubing before entering the reactor (specifically for 7 minutes) before switching to the target liquid flow rate (controlled by the Isco pump). The contents of the gas-liquid separator were drained after 10

minutes. The reaction product was drained through the liquid collector every 1 h, and the collected mass was weighed.

Qualitative GC/MS Analysis

The qualitative analysis of product samples was performed on a single quadrupole (Agilent 5977) GC/MSD equipped with a HP 5 MS column (30 m x 0.32 mm i.d x 0.25 μm), and a Shimadzu GCMS (QP2010) equipped with Agilent DB-FFAP column (30 m x 0.32 mm i.d x 0.25 μm) column. The use of polar and non-polar columns improves the identification of species in the product streams. The method used for analysis includes 250 °C ion source temperature, m/z between 25 and 500, and method (40 ^{o}C/1 min hold, 240 ^{o}C at 10 ^{o}C/min ramp/3 min hold). The data from the GCMS analysis were loaded onto AMDIS software containing an updated NIST database and quality identification was conducted by relying on purity level and matching percentage. The commercially available compounds identified in product streams at significant concentrations were injected into GCMS for the confirmation of retention times. The measured *m/z* spectra of individual compounds match the reference NIST database spectra and are depicted in **Figures S3-S6**. A sample labeled chromatogram of the reaction product stream, using the AMDIS/NIST reference database, is shown in **Figure S7**.

Supplementary HPLC Analysis

Considering that most of the HAs used in this work have low vapor pressure, GC analysis was deemed non-ideal. Thus, the quantitative analysis of HAs feeds and products were analyzed using a Shimadzu HPLC equipped with a Bio-Rad Aminex HPX87-H column (300 mm x 7.8 mm id), connected to both dual ultraviolet (UV at 210 and 250 nm) and refractive index (RID) detectors. The HPLC method used was a mobile phase of 5mM H_2SO_4 in deionized water, 0.5 mL/min, 50

^{o}C oven temperature, and 180 min analysis time for each product sample injection. The injection volume used for both calibration of standard compounds and products was 5 μL, and the response factor is shown in **Table S3**. As the HPLC is very stable and reproducible, the external standard method of calibration was employed. The calibrated curves for each compound resulted in high linearity (R^2 value of ≥ 0.99). The HPLC peaks for the real HA feedstock and sample product (optimum) are shown in **Figure S8**.

Supplementary GC Analysis I

During sample analysis of the realistic HAs feed and products in the HPLC, one hydroxy acid peak overlapped with the propionic acid product peak at a retention time of 20.7 min (HPLC). Thus, the analysis of the reaction product stream was additionally conducted in an Agilent GC 7890B equipped with 2 switching valve systems connected to an Ultra-inert DB Wax column (30 m x 0.25 mm i.d x 0.25 μm) with flame ionization detector, and a PoraBOND U (25 m x 0.32 mm x 0.25 μm) column connected to a thermal conductivity detector (TCD). A typical method includes keeping the oven temperature at 35 ^{o}C for 1 min followed by 5 ^{o}C/min ramping to 180 min (held for 2 minutes) and finally 20 ^{o}C/min ramping to 250 ^{o}C (held for 2 minutes). The response factor of standard compound calibration using 1,4 dioxane as an internal standard is shown in **Table S4**. The FID signal was calibrated using commercially available representative standard compounds to achieve near-perfect linear calibration curves for individual species (R^2 ≥ 0.998). The sample chromatogram of the product stream injected (1 uL injection) in GC is shown in **Figure S9**.

Supplementary GC Analysis II

The gas bag collected during reactions was analyzed in a 7890 Agilent gas chromatography equipped with 3 columns (MolSieve for the detection of light gases, PoraBOND U for the detection

of CO_2, and CP-Wax for the detection of alkanes/alkenes and aromatics) and three detectors (two TCDs and one FID), being controlled by 3 sampling valves for the front, back and auxiliary detectors. A rigorous calibration of more than 50 compounds consisting of light alkanes, oxygenates, CO_2, and CO was conducted, and the RF for relevant individual compounds is shown in **Table S5**. The sample chromatograms for the front, back, and auxiliary detectors are shown in **Figures S10-S12** respectively. The HPLC and integrated GC data were then used to calculate the molar composition of each sample, the conversion, and the selectivities of products.

Catalyst Characterization

Ammonia Temperature Programmed Desorption (TPD): The acid site density of the supported catalysts was estimated using NH_3-TPD in Autochem II 2920 (Micromeritics), equipped with a TCD detector. About 80 mg of sample was loaded into a quartz wool-packed sample tube and pretreated in a flowing 30 mL/min He at 40 ^oC for 30 minutes. This was followed by heating the sample to 250 ^oC (10 ^oC/min ramp rate) and reducing it at this temperature for 2 h. At 250 ^oC, the sample was flushed with 50 mL/min He for 30 min and finally cooled to 100 ^oC. Finally, 3000 ppm NH_3/He flowing at 50 mL/min was adsorbed on the sample for 1 h. The adsorbed ammonia was removed by flushing the system with He, followed by heating the sample from 100 ^oC to 600 ^oC (10 ^oC/min) under He flow (50 mL/min) during which the desorbed ammonia was detected by the MS.

H_2 Pulse Chemisorption: This was conducted using an Autochem II 2920 (Micromeritics, USA) equipped with a TCD detector. About 80 mg of powdered catalyst was loaded in a U-shaped quartz tube and Ar (30 mL/min) was allowed to flow over the sample for 30 min. This was followed by reducing the sample to 10%H_2/Ar (50 mL/min) at 400 ^oC for 2 h. The reduced sample was then

flushed with Ar at the same temperature for 1 h before cooling to 40 °C. Pulses of 10% H_2/Ar were introduced to the sample repeatedly until the TCD peak signal remained constant between pulses.

X-ray Diffraction: This was performed at room temperature on a PANalytical XPert PRO Alpha-1 (Malvern Panalytical) diffractometer using Cu Kα radiation in the 2θ range from 20° to 70°, at a step size of 0.0167°.

N_2 Physisorption: A Tristar II 3020 (Micromeritics) sorption analyzer was used to collect isotherms at -196 °C using ~100 mg of catalyst samples. Degassing under vacuum was performed at 180 °C for 3 h before measurements.

Inductively coupled plasma optical emission spectroscopy (ICP-OES): This method was used to determine the loadings of Pd and Nb before and after the hydrodeoxygenation of the model mixture of hydroxy acids.

Calculation Formulae

$$Carbon\ recovery = \frac{\Sigma n_{HC,\,offline} + \Sigma n_{oxygenates} + n_{CO2} + n_{CO}}{n_{HA,\,consumed}}$$

n = number of moles of carbon atoms, HA = hydroxy acid

$$Reaction\ products\ \ C\_mol_i = \frac{A_i}{RF\_H} * number\ of\ carbon\ atoms$$

RF_H is the response factor derived from HPLC injection of standard compounds (**Table S3**)

A = Peak area of species "i", C_mol_i = mol of carbon in species "i"

$$Reaction\ products\ \ C\_mol_i = \frac{1}{RF\_G} * \frac{A_i}{A_{istd}} * mol_{istd} * number\ of\ carbon\ atoms$$

RF_G is the response factor derived from GC injection of standard compounds (**Table S4**)

A = Peak area of species "i", C_mol_i = mol of carbon in species "i", istd = 1,4 dioxane

$$HAs\ conversion = \frac{mol_{carbon\ feed\_in} - mol_{carbon\_feed\_out}}{mol_{carbon\ feed\_in}} * 100$$

$$Degree\ of\ deoxygenation = \frac{mol_{O2\ in\ feed} - mol_{O2\ in\ product}}{mol_{O2\ in\ feed}} * 100$$

$$Normalized\ Product\ distribution = \frac{mol_{carbon\_i}}{\sum mol_{carbon\_quantified}} * 100$$

$$Rate\ of\ HDO = \frac{(C_{O2_{infeed}} - C_{O2\ in\ product})mol}{g} * \frac{m_{sample\ collected\ (g)}}{Accumulation\ time\ (h)} * \frac{1}{Catalysts\ mass\ (g)}$$

XRD crystallite domain size calculation: Calculated using Scherrer equation:

$$L = \frac{K\lambda}{DCos(\theta)}$$

K= Constant (0.94), D = Full Width at Half Maximum (FWHM), λ = Wavelength of X-ray (0.154 nm), θ = Peak angle, and L = Crystallite size. The FWHM was estimated using the Gaussian nonlinear peak fitting tool contained in OriginPro.

II. Supporting Tables

Table S1. Model mixed hydroxy acids (HAs) for catalytic upgrading. FA = Formic acid, AA = Acetic acid, Glyc A = Glycolic acid, LA = Lactic acid, Gluc A = Gluconic acid, SA = Succinic acid, MA = Malic acid, DMPA = 2,2-bis(hydroxymethyl) propionic acid

* The model acids are representative of the acids present in real kraft BL, which are divided into 9 classes (see Table S2) based on their number of -OH and -COOH groups.

Class*	-OH	-COOH	HA (abbrev.)	Formula	Conc (g/L)	wt %	mol/L	Carbon Number
1	0	1	FA	CH_2O_2	52.3	4.20	0.801	1
			AA	$C_2H_4O_2$	20.8	1.67	0.229	2
2	1	1	Glyc A	$C_2H_4O_3$	23.9	1.92	0.208	2
			LA	$C_3H_6O_3$	31.8	2.54	0.233	3
3	2	1	DMPA	$C_5H_{10}O_4$	46.5	3.72	0.229	5
4 - 5	5	1	Gluc A	$C_6H_{12}O_7$	56.0	4.48	0.188	6
6	0	2	SA	$C_4H_6O_4$	2.5	0.23	0.014	4
7 - 9	1	2	MA	$C_4H_6O_5$	15.4	1.24	0.076	4
NA			Water	H_2O		80.00		0

Table S2. Composition of kraft black liquor-derived hydroxy acid feed. * Acids are divided into 9 classes based on the number of -OH and -COOH groups. a = erythro- and threo- isomers, b = α- and β- isomers. Other components: lignin (0.08 wt%), Na_2SO_4 (0.04 wt%), methanol (0.16 wt%).

Class*	# OH	COOH	Organic acids	Formula	Conc g/L	wt%	mol/L	Carbon Number
**Volatile Acids**					**17.86**	**1.77**		
1	0	1	Formic acid (FA)	CH_2O_2	13.50	1.34	0.3154	1
			Acetic acid (AA)	$C_2H_4O_2$	4.36	0.43	0.0780	2
**Hydroxy monocarboxylic acids**					**57.16**	**5.67**		
2	1	1	Lactic acid (LA)	$C_3H_6O_3$	9.89	0.98	0.1180	3
			Glycolic acid (GA)	$C_2H_4O_3$	5.15	0.51	0.0728	2
			2-Hydroxybutanoic acid	$C_4H_8O_3$	5.33	0.53	0.0550	4
			2-Hydroxypentenoic acid	$C_5H_8O_3$	0.02	0.00	0.0002	5
			2-Hydroxypentanoic acid	$C_5H_{10}O_3$	0.79	0.08	0.0072	5
			4-Hydroxybutanoic acid	$C_4H_8O_3$	0.24	0.02	0.0025	4
3	2	1	2-Methylglyceric acid	$C_4H_8O_4$	0.73	0.07	0.0054	6
			Glyceric acid	$C_3H_6O_4$	0.08	0.01	0.0008	3
			3-Deoxytetronic acid	$C_4H_8O_4$	0.89	0.09	0.0080	4
			2-Deoxytetronic acid	$C_4H_8O_4$	0.48	0.05	0.0043	4
			2,5-Dihydroxypentanoic acid	$C_5H_{10}O_4$	9.57	0.95	0.0767	5
4	3	1	Xylo-isosaccharic acid (XISA)	$C_5H_{10}O_5$	1.61	0.16	0.0096	6
			3-Deoxypentonic acid[a]	$C_5H_{10}O_5$	2.58	0.26	0.0185	5
			3,6-Dideoxyhexonic acid	$C_6H_{12}O_5$	0.62	0.06	0.0037	6
			3,4-Dideoxyhexonic acid	$C6H_{12}O_5$	0.82	0.08	0.0049	6
5	4	1	Galactometasaccharinic acid[b]	$C6H_{12}O_6$	0.82	0.08	0.0049	6
			Gluco-isosaccharinic acid (GISA)[b]	$C_6H_{12}O_6$	17.54	1.74	0.1047	6
**Hydroxy dicarboxylic acids**					**3.63**	**0.36**		
6	0	2	Succinic acid (SA)	$C_4H_6O_4$	1.18	0.12	0.0107	4
7	1	2	C-Methyltartronic acid	$C_4H_6O_5$	0.24	0.02	0.0019	4
			Tartronic acid	$C_3H_4O_5$	0.26	0.03	0.0023	3
			Citramalic acid	$C_5H_8O_5$	0.12	0.01	0.0009	5
			Malic acid (MA)	$C_4H_6O_5$	0.51	0.05	0.0041	4
			2-Hydroxyglutaric acid	$C_5H_8O_5$	0.76	0.08	0.0055	5
8	2	2	3,4-Dideoxyhexaric acid	$C_6H_{10}O_6$	0.36	0.04	0.0022	6
9	3	2	(2,3-Dihydroxypropyl) tartronic acid	$C_6H_{10}O_7$	0.20	0.02	0.0020	4
**Total Organic acids**					**78.65**	**7.80**		
			Water	H_2O		**91.92**		

Table S3. Response Factors from HPLC (Bio-Rad Aminex HPX87-H column).

Species	RT (min)	RF	Species	RT (min)	RF
Formic acid	16.6	1.50E+09	Hydroxyacetone	20.2	3.00E+09
Acetic acid	17.7	2.00E+09	2-butanone	33.1	3.00E+09
Glycolic acid	14.2	4.00E+09	Methyl-cyclopentenolone	38.1	6.00E+09
Lactic acid	14.7	5.00E+09	3 pentanone	43.2	5.00E+09
Malic acid	11.1	7.00E+09	4 heptanone	72.9	6.00E+09
Succinic acid	13.3	7.00E+09	cyclohexanone	56.2	7.00E+09
2,5 dimethylol propionic acid	14.2	8.00E+09	2-methyl cyclopentanone	52.1	6.00E+09
Gluconic acid	10.3	1.20E+10	3-methyl cyclopentanone	54.2	6.00E+09
2-Hydroxybutanoic acid	12.8	6.00E+09	Acetaldehyde	21.8	2.00E+09
3-Deoxytetronic acid	15.5	7.00E+09	Isobutyraldehyde	33	4.00E+09
2,5-Dihydroxypentanoic acid	15.1	8.00E+09	Levulinic acid	18.5	6.00E+09
Xylo-isosaccharic acid (XISA)	10.3	1.00E+10	Gamma-valerolactone	35.5	5.00E+09
3-Deoxypentonic acid	12.1	8.00E+09	Methanol	25.6	3.00E+09
3,4-Dideoxyhexonic acid	8.4	1.60E+10	Ethanol	22.5	3.00E+09
Galactometasaccharinic acid	9.7	1.00E+10	Isobutanol	44.1	4.00E+09
Gluco-isosaccharinic acid (GISA)	13.1	1.50E+10	1-butanol	40.6	4.00E+09
4-Hydroxybutanoic acid	9.4	7.00E+09	1-pentanol	58.4	5.00E+09
2-Methylglyceric acid	8.6	7.00E+09	Propionic acid	20.7	3.00E+09
Glyceric acid	8.1	7.00E+09	n-butyric acid	23.34	5.00E+09
3-Deoxytetronic acid	15.5	6.00E+09	i-butyric acid	25.4	5.00E+09
2-Deoxytetronic acid	16.2	6.00E+09	e methyl Butyric acid	27.1	5.00E+09
C-Methyltartronic acid	22.01	7.00E+09	Methacrylic acid	27.8	5.00E+09
Tartronic acid	19.57	7.00E+09	Pentanoic acid	36.4	6.00E+09
Citramalic acid	19.12	8.00E+09	Hexanoic acid	51.5	7.00E+09
2-Hydroxyglutaric acid	17.1	8.00E+09	4 methyl octanoic acid	36.2	7.00E+09
2-hydroxypentanoic acid	20.7	7.00E+09	Acetone	26.5	2.00E+09
Lactic acid ethyl ester	49.1	6.00E+09	Phenol	135.1	5.35E+09

The commercially available organic acids in the realistic kraft-BL-derived hydroxy acids constitute ~ 52 % of the total acids and were accurately calibrated with an R^2 value ≥ 0.995. About 44 % of the acids are not commercially available for calibration. Hence, the response factors were approximated based on the class categories (# OH and # COOH) shown in Table S1. The retention times were assigned by correlating peak heights to the concentrations of these species in the mixture. Specifically, the retention times of Gluco-isosaccharinic acid (22 %), 2 hydroxybutanoic acid (7 %), 2,5 dihydroxypentanoic acid (12 %), 2-deoxypentonic acid (4 %) were easily assigned based on their relatively high concentrations in the realistic acid feedstock. An alternative estimation of conversion was designed by performing calibration using the concentration of individual acids in the mixture. A constant weight (0.1g) was added into 5 different vials containing 1 mL, 3 mL, 6 mL, 10 mL, and 15 mL DI water to obtain a calibration curve.

Table S4. Response Factor from GC (Ultra inert DB Wax column).

Species	Retention Time	Response Factor
2-hexene	1.97	0.21
Acetic acid	8.4	0.43
Propionic acid	9.58	0.9
n-butyric acid	10.69	1.27
Pentanoic acid	11.98	1.67
Hexanoic acid	13.19	2.07
Acetaldehyde	1.67	0.45
3-Butenoic acid	11.97	1.15
3-pentanone	3.25	1.71
Isobutyraldehyde	2.02	0.99
Methacrylic acid	11.37	1.22
Levulinic acid	17.71	0.93
p-cresol	15.58	1.21
m-xylenol	15.92	1.09

Table S5. Response Factor from GC (CP-Wax, PoraBond U, MolSieve columns).

Species	Aux	Back	Front
Carbon monoxide	1.9E-06	0	0
Carbon dioxide	0	0	6.44E-07
Methane	4.78E-06	4.5E-08	0
Ethylene	0	4.5E-08	5.45E-07
1-Propylene	0	4.5E-08	4.35E-07
Dimethyl Ether	0	8.89E-08	5.54E-07
1-Butene	0	2.06E-08	2.87E-07
Pentane	0	2.06E-08	2.99E-07
1-Pentene	0	1.8E-08	2.99E-07
Hexane (1)	0	1.8E-08	2.46E-07
Hexane (2)	0	1.8E-08	2.46E-07
Hexane (3)	0	1.8E-08	2.46E-07
1-Hexene	0	1.86E-08	2.46E-07
Hexane (4)	0	1.8E-08	2.46E-07
Heptane	0	1.46E-08	1.12E-07
Benzene	0	2.06E-08	0
Toluene	0	1.44E-08	0
Ethyl Benzene	0	1.35E-08	0
p-Xylene	0	1.3E-08	0
m-Xylene	0	1.35E-08	0

Table S6. The carbon selectivity for the conversion of model gluconic acid at different Pd loadings on Nb_2O_5 and carbon supports (230 ^oC, 60 bar, 2.85 h^{-1}, 50 mL/min H_2 co-flow). The data are not normalized.

		mol %				
**Group Name**	**Species**	**0.1Pd/Nb**	**0.25Pd/C**	**0.25 Pd/Nb**	**0.5Pd/Nb**	**1.0Pd/Nb**
Hydroxy acids	Gluconic acid	8.13	7.01	4.95	3.66	1.03
	Glucaric acid	15.17	7.59	5.92	3.94	2.61
	Glycolic acid	3.12	0.22	0.74	0.19	0.15
	Lactic acid	5.34	3.02	2.87	1.58	0.48
	Malic acid	0.50	0.35	0.13	0.08	0.02
Esters	Gluconic acid ɣ-lactone	4.69	2.82	7.03	7.94	8.04
	Gluconic acid d-lactone	3.71	1.32	5.21	5.98	6.87
	Gamma valerolactone	2.45	1.01	3.88	4.24	4.42
	Gamma heptalactone	1.48	0.32	0.75	1.30	2.05
	Ethyl acetate	3.05	3.42	3.11	2.42	3.66
Aldehyde	Acetaldehyde	0.84	1.21	0.98	1.05	1.29
Carboxylic acids	Formic acid	0.60	1.56	1.42	1.15	1.40
	Acetic acid	5.34	5.41	8.19	8.60	9.63
	Propionic acid	3.12	5.29	6.90	4.34	2.45
	Butyric acid	0.84	0.97	0.91	0.83	0.59
	Hexanoic acid	0.09	0.15	0.12	0.43	0.48
Keto-acids	Levulinic acid	7.81	13.96	15.84	14.91	16.07
Ketones	Acetone	1.09	0.06	1.38	1.46	1.30
	3-pentanone	0.09	0.01	0.23	0.25	0.31
	Cyclohexanone	1.69	1.95	3.38	3.50	3.32
Alcohols	1 butanol	1.12	3.28	1.17	0.83	0.79
	1,3,5 benzenetriol	0.49	1.43	0.23	0.31	0.28
	Sorbitol	1.01	11.08	0.94	0.53	0.32
	Xylitol	2.48	1.76	3.10	7.55	10.03
COx	CO_2	3.01	6.12	3.57	3.99	4.87
	CO	0.01	0.04	0.02	0.03	0.03
Hydrocarbons	2-hexene	3.12	4.56	2.76	4.76	6.01
	Methane	0.13	0.42	0.21	0.19	0.17
	Light olefins	0.11	0.22	0.18	0.21	0.23
Total Carbon		80.65	86.49	86.13	86.25	88.91

Nb = Nb_2O_5

Table S7. The product selectivity for the conversion of mixed model HA feed at different temperatures over 0.25%Pd/Nb_2O_5 and 0.25%Pd/C (60 bar, 3.1 h^{-1}, 50 mL/min H_2 co-flow). Other compounds detected in trace amounts are acids (sorbic acid, heptanoic acid), Esters (Butyric acid 2-ethyl, hexanoic acid 2-hexenyl ester), ketones (hydroxy-2 butanone).

		Product Distribution (%)			
Group	Species	240 °C [a]	260 °C [b]	260 °C [a]	280 °C [a]
C_2 - C_6 acids	Acetic acid	2.91	0.51	2.36	1.96
	Propionic acid	2.35	1.23	3.99	5.23
	n-butyric acid	0.74	0.78	0.59	2.09
	iso-butyric acid	1.16	1.48	2.73	3.88
	Methacrylic acid	1.58	1.63	1.13	0.8
	Pentanoic acid	0.25	0.77	0.62	1.08
	Hexanoic acid	0.12	0.45	0.47	0.62
Aldehydes	Acetaldehyde	0.21	0.75	0.57	1.24
	Isobutyraldehyde	1.77	11.31	5.36	8.92
Alcohols	isobutanol	0.2	1.97	0.73	1.19
	Ethanol	0.01	0.37	0.08	0.02
	Methanol	0.08	0.01	0.11	0.02
	1-pentanol	0.02	n/a	0.04	0.07
	Sorbitol	n/a	9.12	n/a	n/a
	Xylitol	n/a	1.45	n/a	n/a
Enol/Aromatics	p-cresol	6.59	1.12	4.28	1.09
	1,3,5 benzenetriol	6.06	2.23	4.01	0.84
	m-xylenol	2.91	0.05	1.82	0.67
Esters/Anhydrides	Ethyl propionate	2.03	0.23	1.62	0.08
	Ethyl acetate	0.76	0.04	0.21	0
	2-pentyl acetate	1.23	0.87	0.09	0.12
	Lactic acid ethyl ester	10.36	0.94	4.91	1.05
	Succinic anhydride	2.69	0.93	2.01	0.81
	Gluconic acid δ-lactone	5.1	1.06	1.95	0.42
	ɣ-valerolactone	2.87	0.64	3.73	7.65
Ketones	Acetone	3.47	1.21	2.05	1.26
	Hydroxyacetone	0.31	0.56	0.19	0.06
	2 methyl cyclopentanone	0.1	1.24	0.91	1.2
	3 methyl cyclopentanone	0.32	0.36	1.19	1.24
	Cyclohexanone	0.34	1.73	1.48	1.58

	Corylone	0.63	2.34	3.72	5.15
Keto-acid	Levulinic acid	6.12	7.2	3.66	1.09
CO_x		21.22	29.21	18.65	21.51
Hydrocarbons		15.35	16.21	25.1	27.11
Total		100	100	100	100

Note. [a] = $0.25Pd/Nb_2O_5$, [b] = 0.25Pd/C. The total conversion of mixed HAs on 0.25 Pd/C is ~ 47% compared to ~ 72 % for $0.25Pd/Nb_2O_5$ catalyst.

Table S8. ICP-OES analyses of $0.25Pd/Nb_2O_5$ (fresh and used), and post-reaction mixture

Samples	Nb	Pd	Pd/Nb
Pd/Nb_2O_5 (Fresh)	42.2%	0.21%	0.005
Pd/Nb_2O_5 (Used)	44 %	0.16%	0.004
Post-reaction solution*	7 ppm	< 1 ppm	n/a

* Product from the hydrodeoxygenation of model mixture of HAs accumulated for 10 h.

III. Supporting Figures

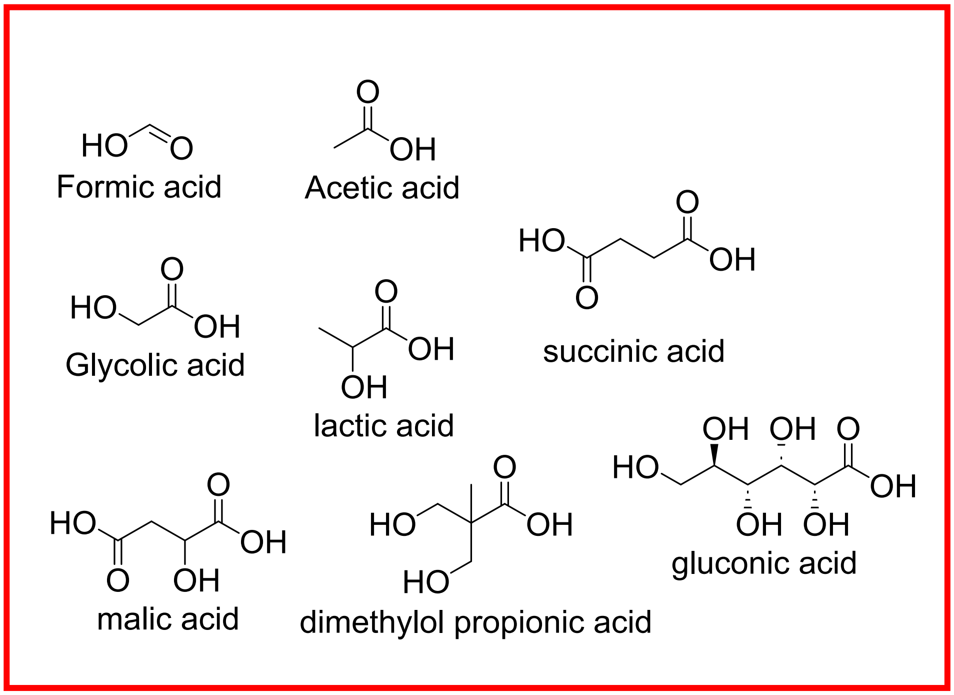

Figure S1. Structures of the individual acids making up the mixed model hydroxy acids used in this work.

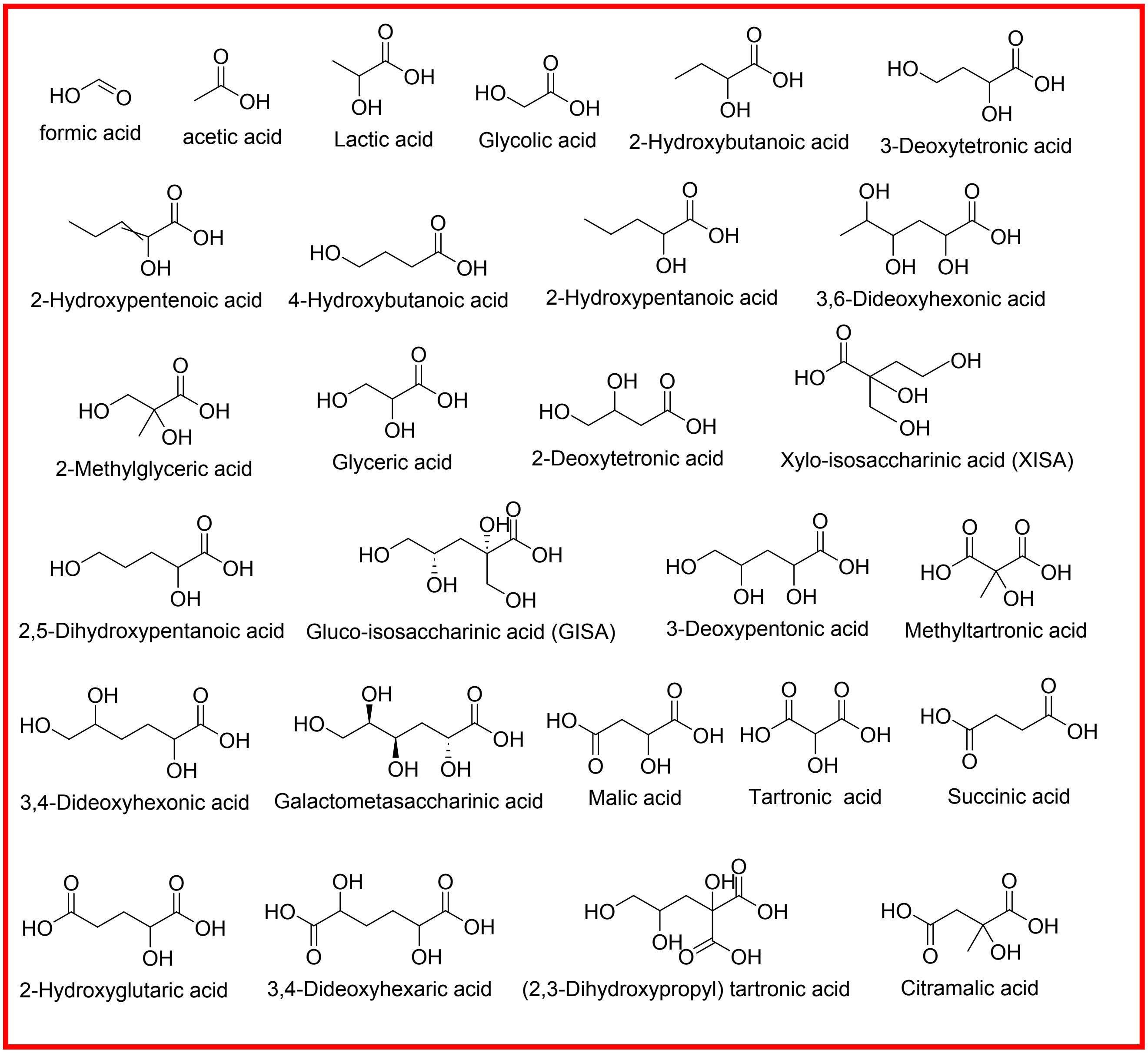

Figure S2. Structures of the individual acids making up the kraft black liquor-derived hydroxy acids used in this work.

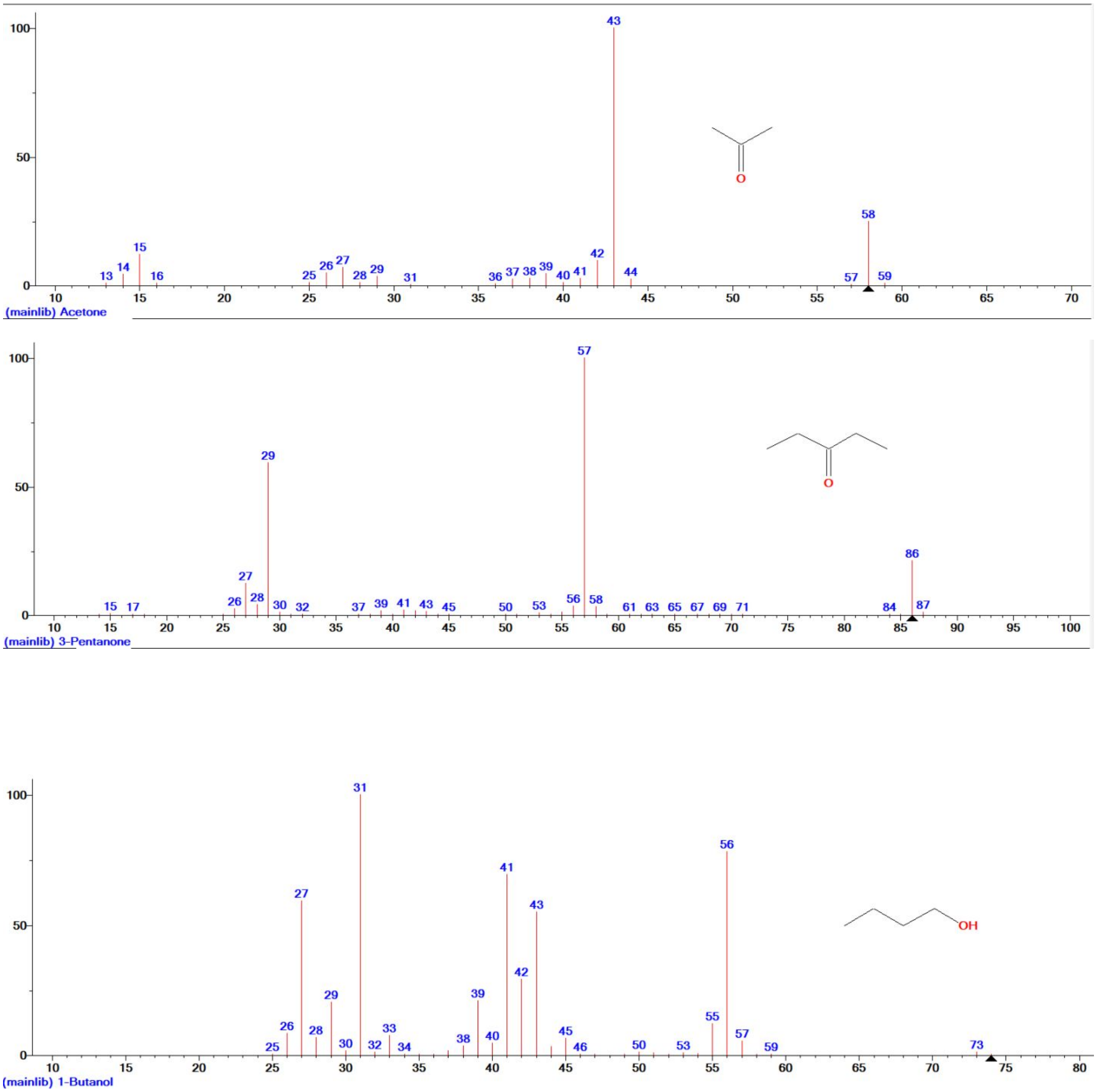

Figure S3. Mass spectra of acetone, 3 pentanone and 1-butanol.

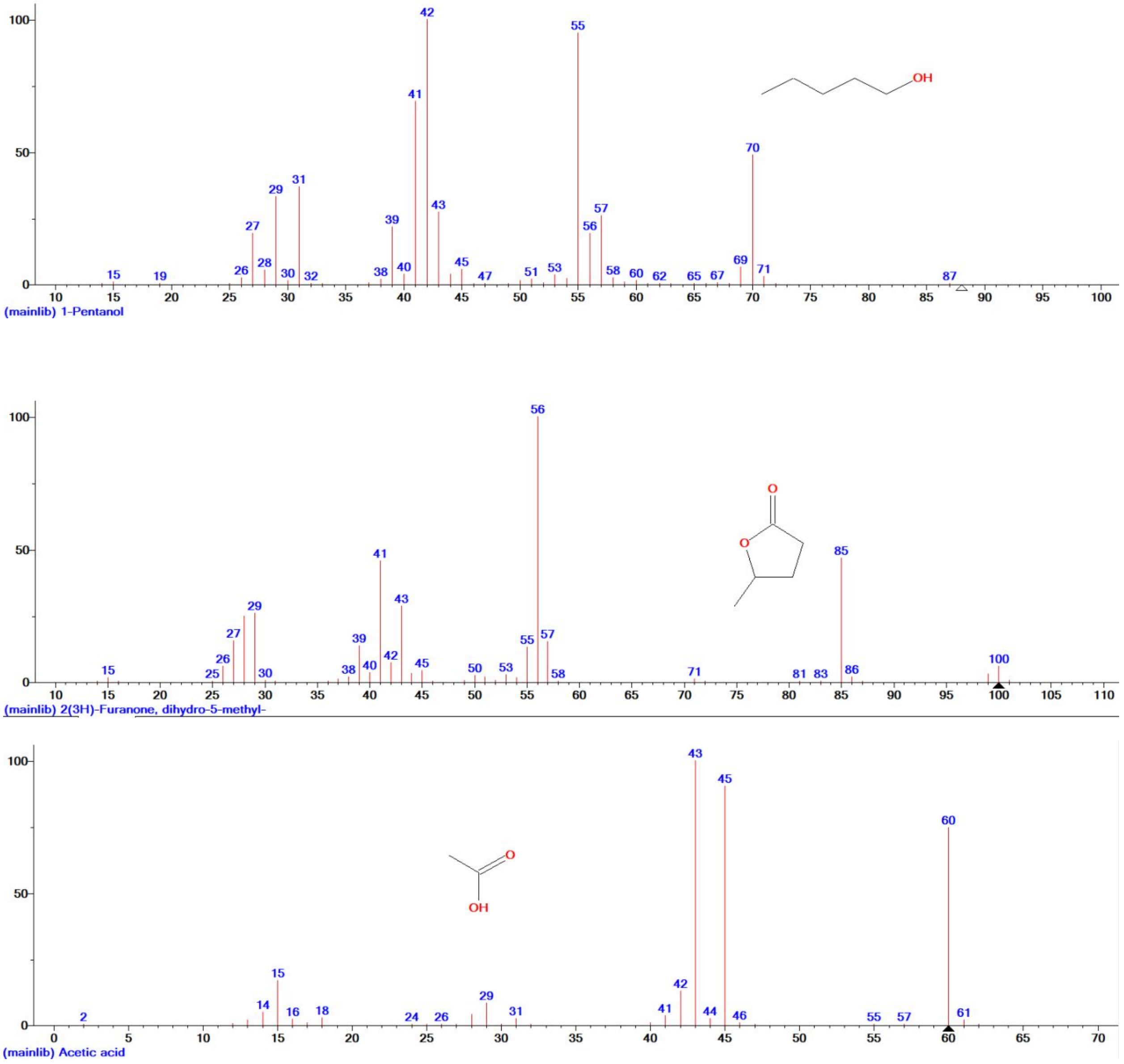

Figure S4. Mass spectra of 1-pentanol, gamma-valerolactone and acetic acid.

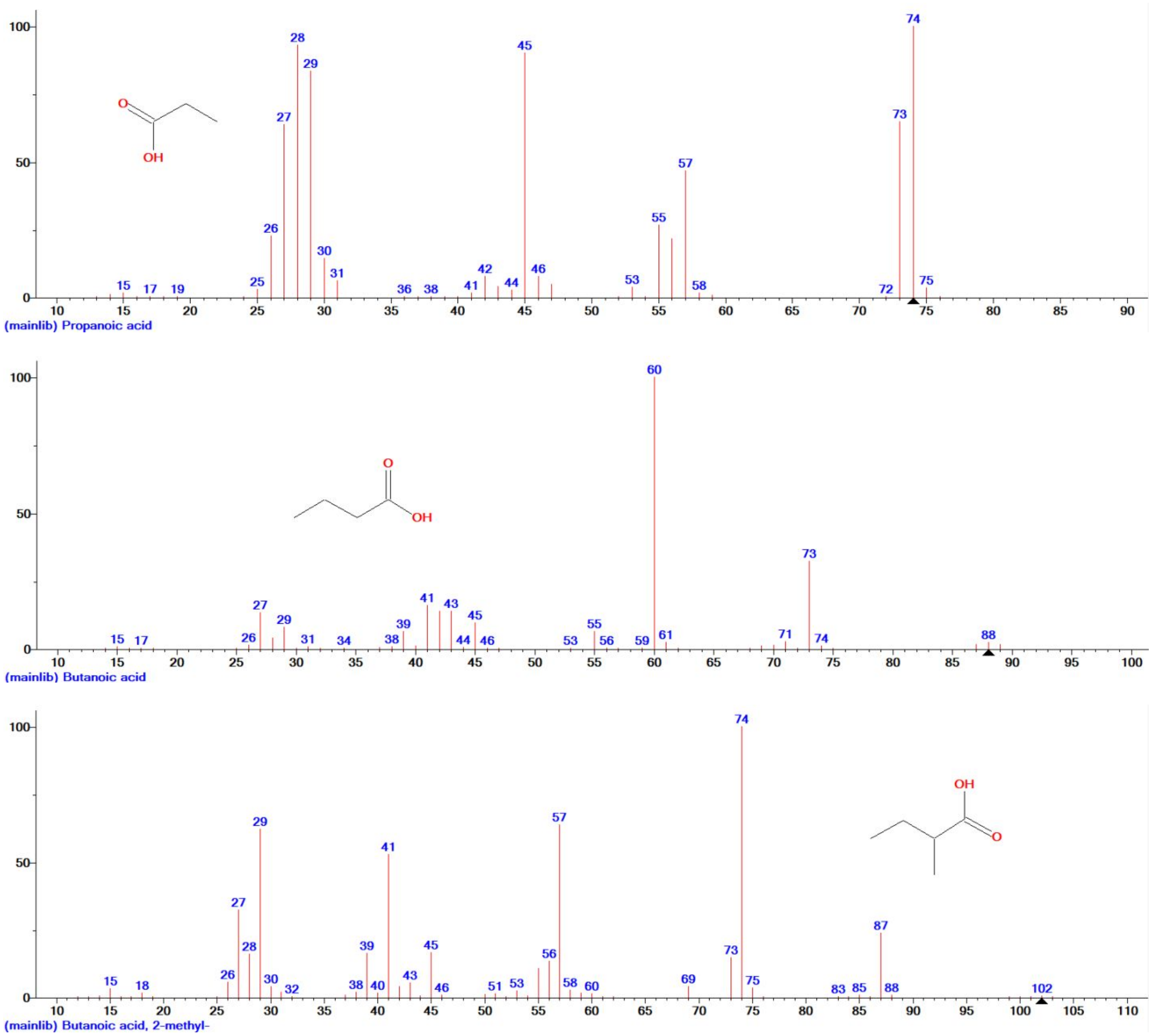

Figure S5. Mass spectra of propanoic acid, butanoic acid and 2-methyl butanoic acid.

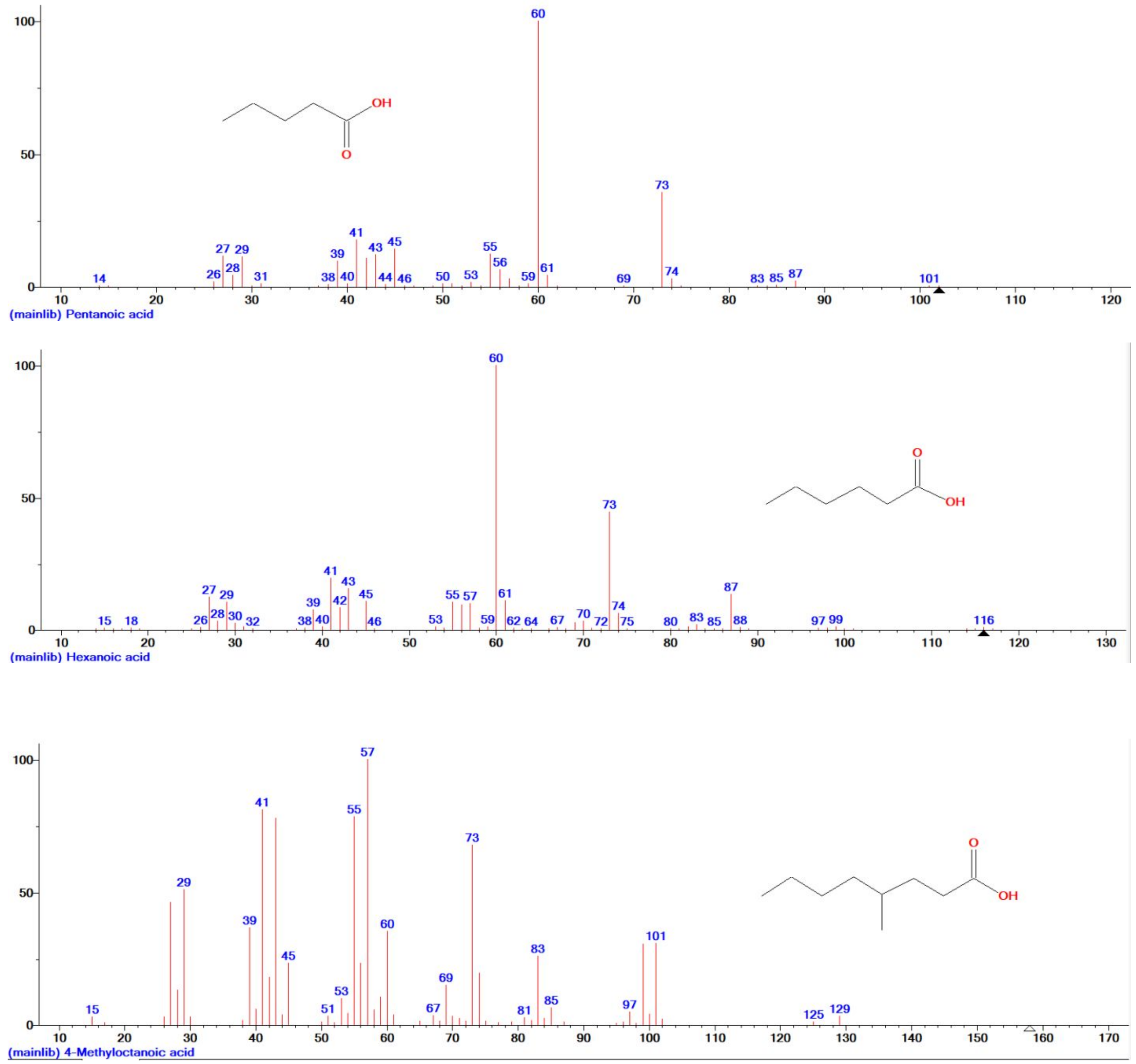

Figure S6. Mass spectra of pentanoic acid, hexanoic acid and 4-methyl octanoic acid.

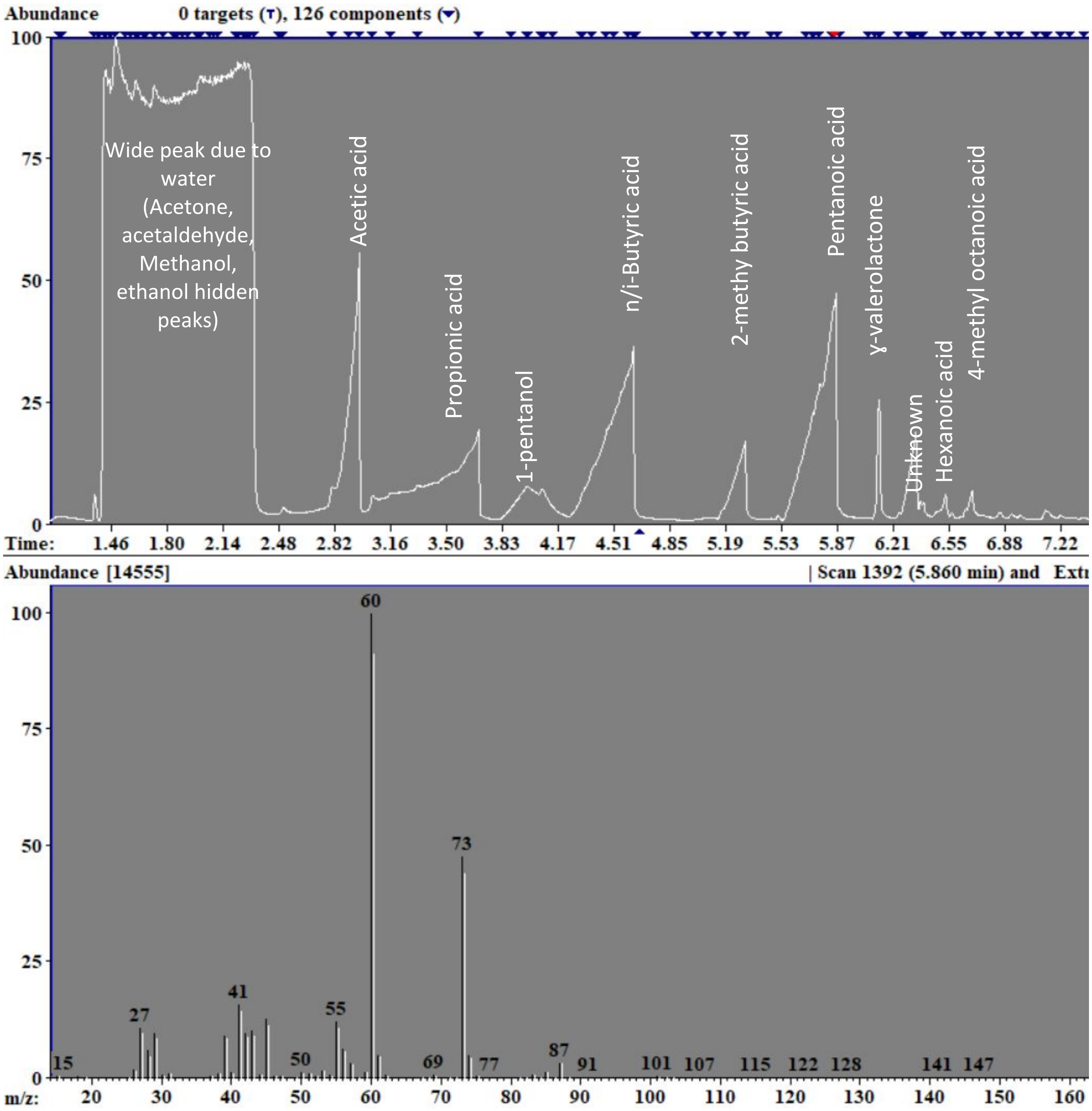

Figure S7. Sample GC/MS chromatogram of product from conversion of the real HA mixture.

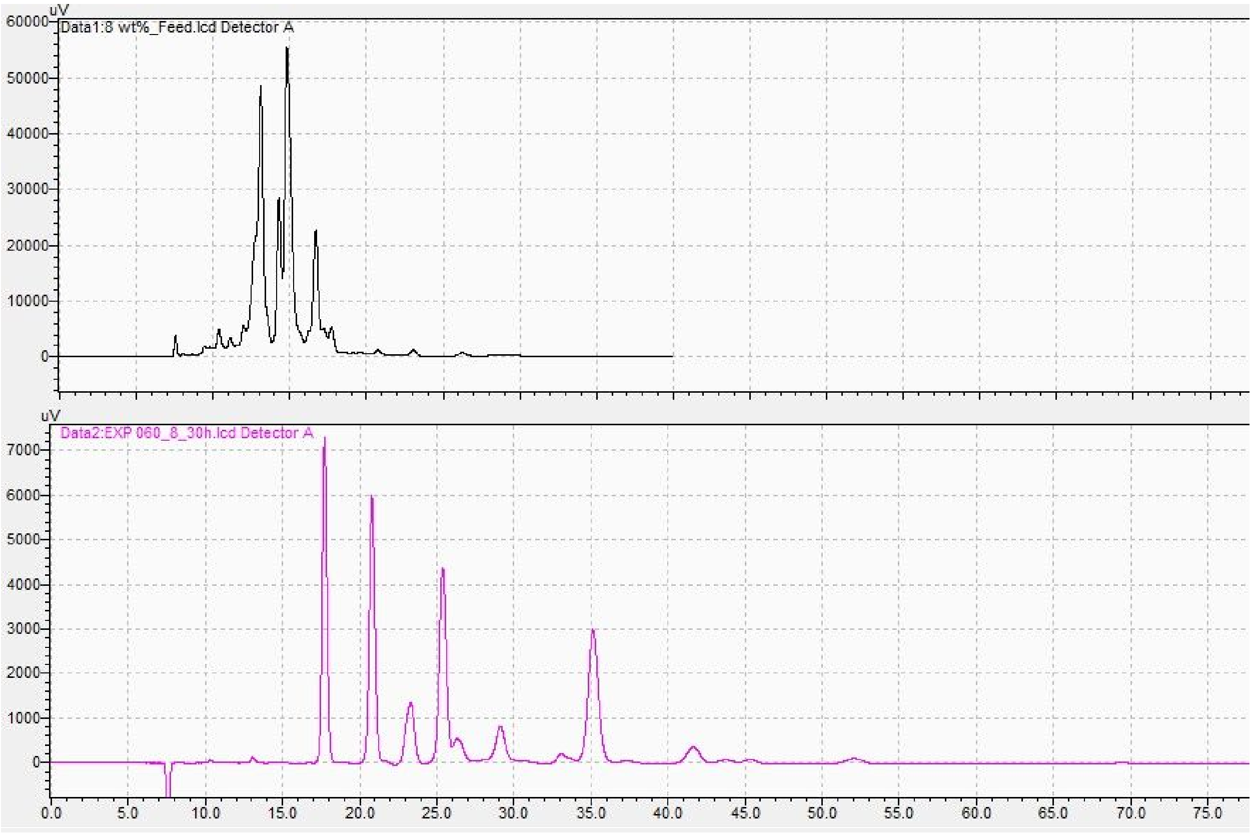

Figure S8. Sample HPLC chromatogram of real HA mixture feed (top) and reaction product (bottom).

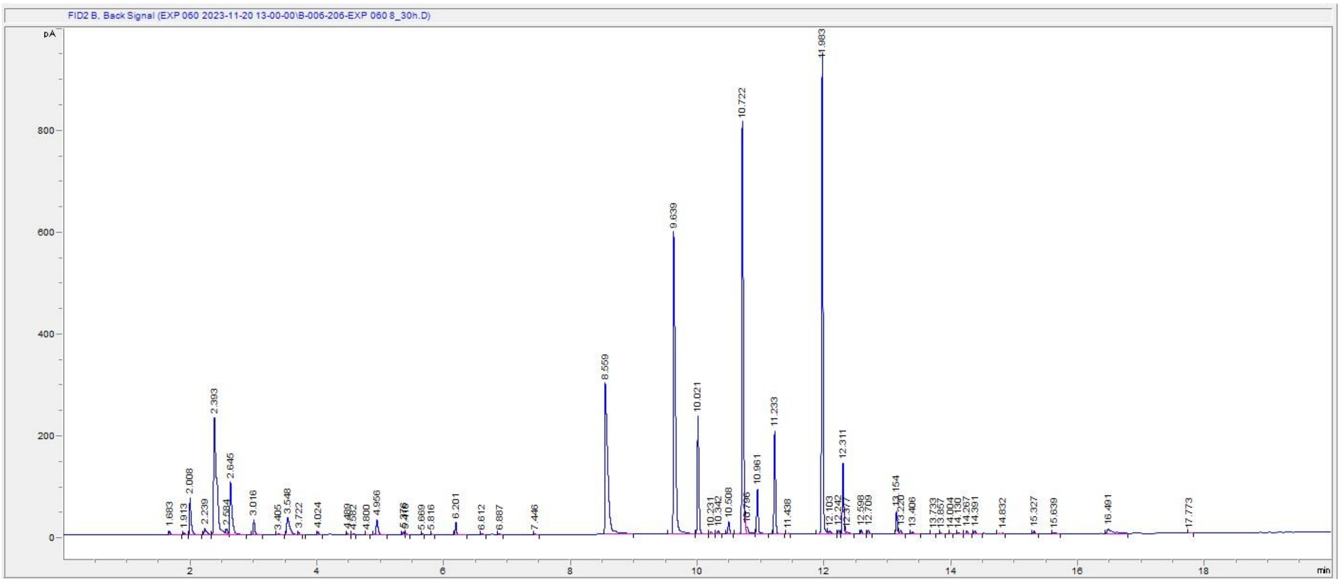

Figure S9. Sample GC chromatogram of reaction products from the conversion of real HA feed (Column: Ultra Inert DB Wax, Detector: FID).

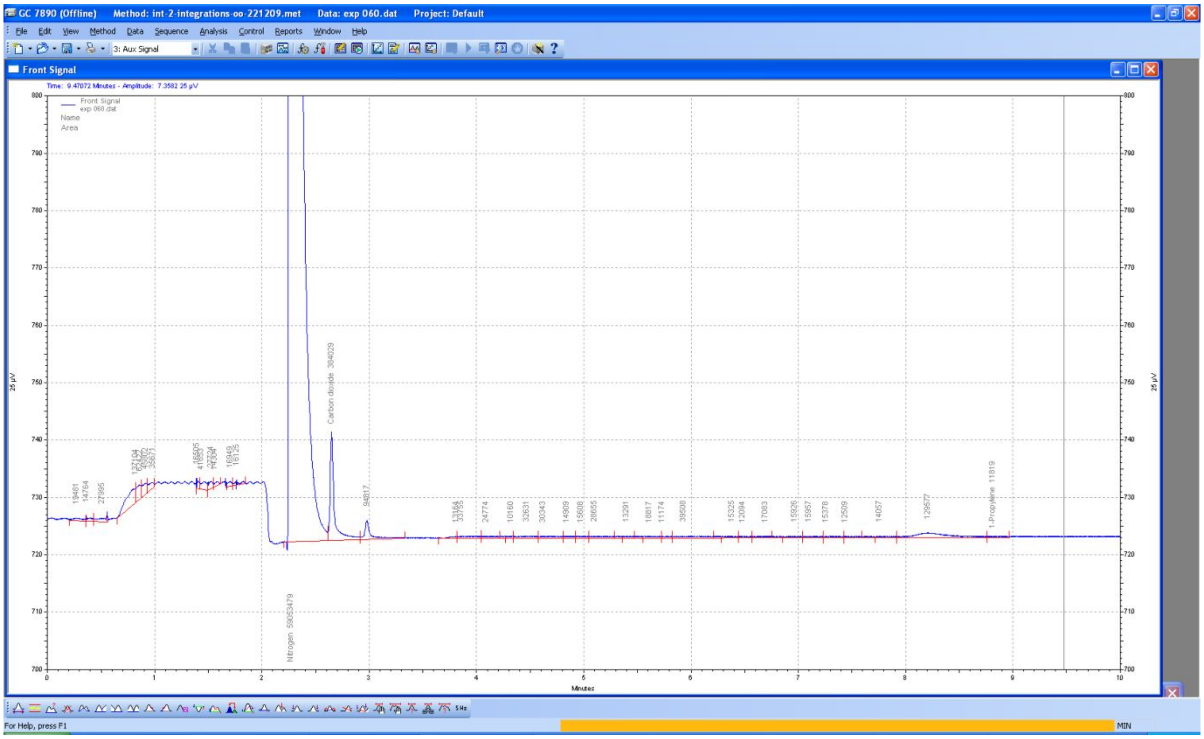

Figure S10. GC chromatogram of vapor products (Detector: TCD).

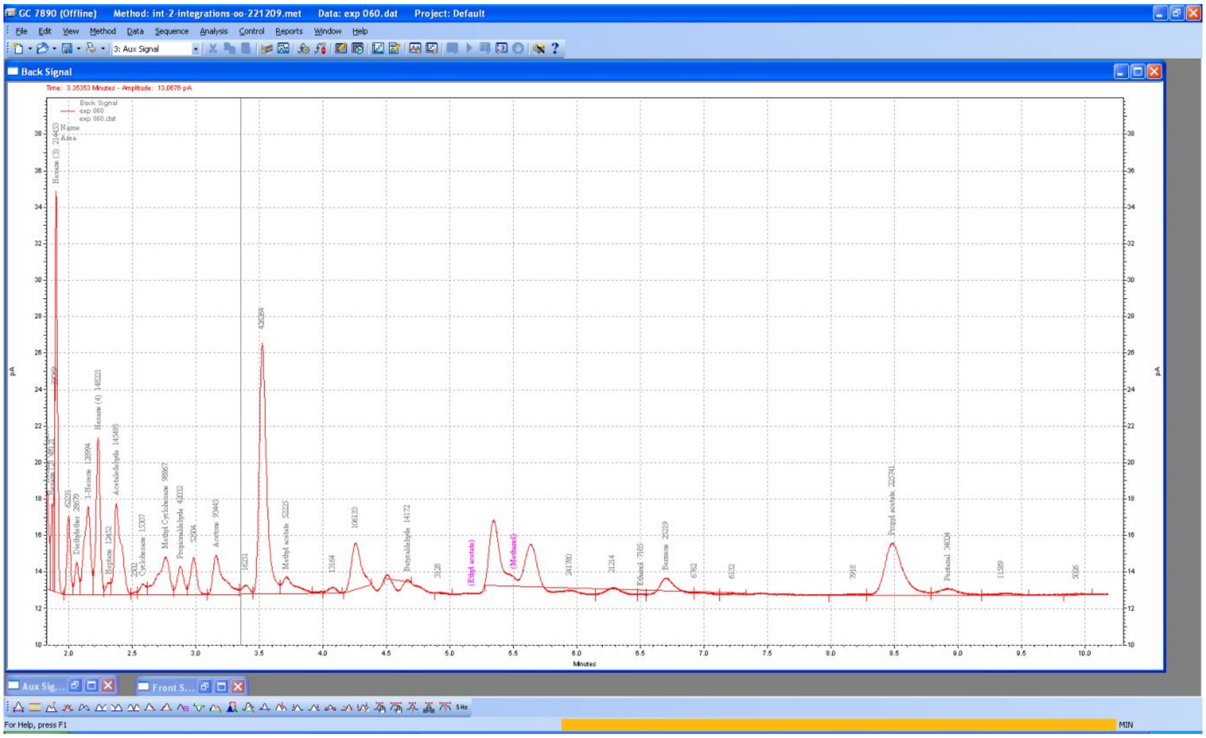

Figure S11. GC chromatogram of vapor products (Detector: FID).

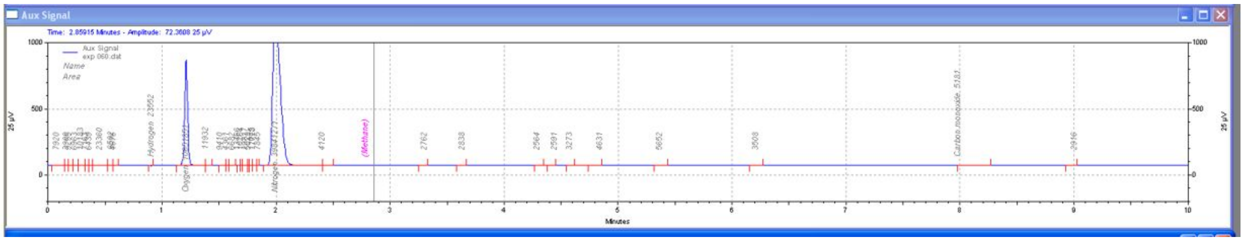

Figure S12. GC chromatogram of vapor products (Detector: TCD).

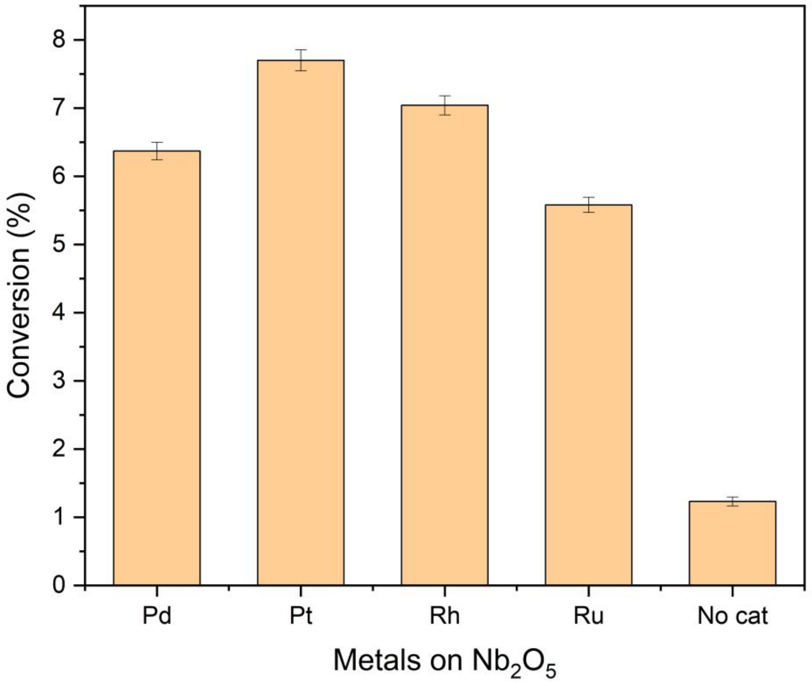

Figure S13. Conversion of model gluconic acid over 0.25% M/Nb_2O_5 (M = Pd, Pt, Rh, and Ru) at low-conversion operating conditions: 150 ^oC, 60 bar, 0.1 mL/min (equivalent to 2.85 h^{-1}), 50mL/min H_2 co-flow. Data were taken after 5 h on-stream. Carbon recovery: 93 – 95 %.

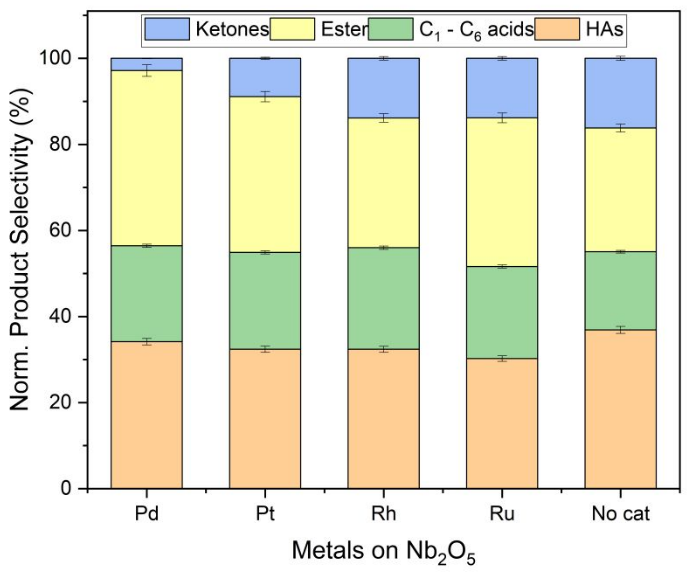

Figure S14. Product selectivity for the conversion of model gluconic acid over 0.25% M/Nb_2O_5 (M = Pd, Pt, Rh, and Ru) at low-conversion operating conditions: 150 ^oC, 60 bar, 0.1 mL/min (equivalent to 2.85 h^{-1}), 50 mL/min H_2 co-flow. Data were taken after 5 h on-stream. HAs represent organic acids containing OH functional groups. Carbon recovery: 93 – 95 %.

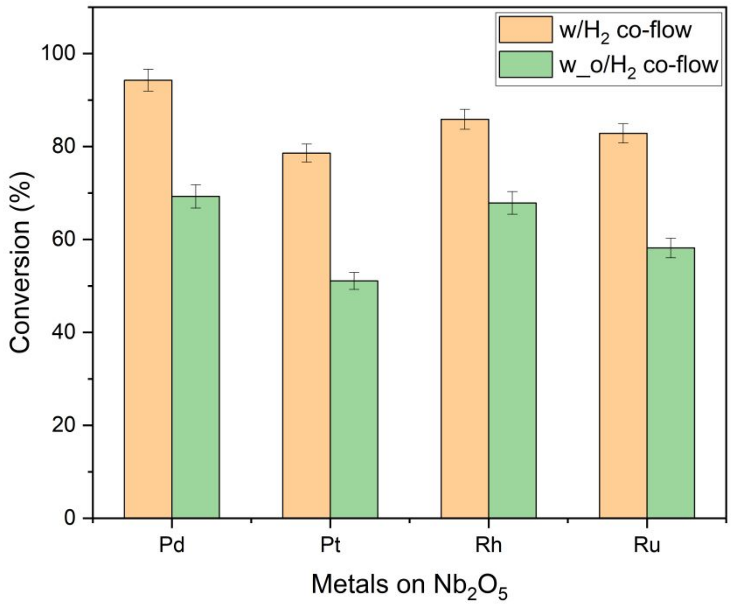

Figure S15. Conversion comparison for model gluconic acid reaction over 0.25%M/Nb_2O_5 (M = Pd, Pt, Rh, and Ru), with and without H_2 co-flow at 230 ^oC, 60 bar, and 0.1 mL/min (equivalent to 2.85 h^{-1}). Data were taken after 5 h on-stream. Carbon recovery: 86 – 89 %.

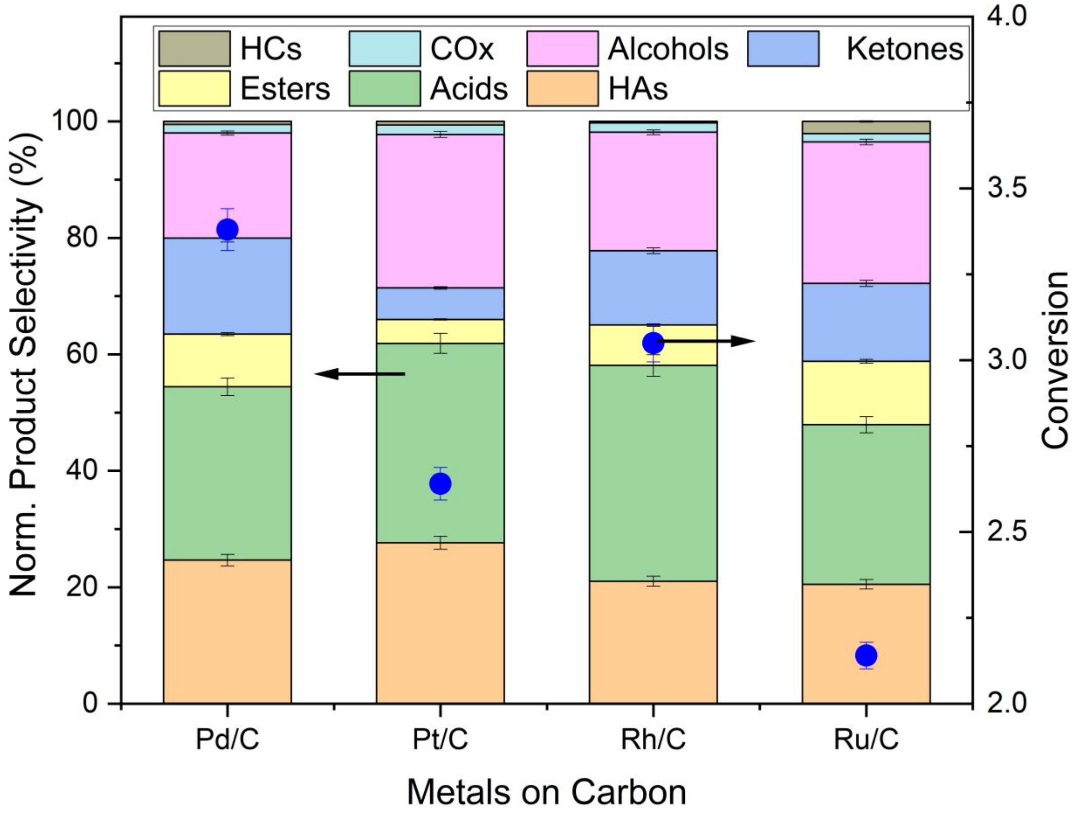

Figure S16. Product selectivity for the conversion of model gluconic acid over 0.25% M/C (M = Pd, Pt, Rh, and Ru) at low-conversion operating conditions: 150 ^{o}C, 60 bar, 0.1 mL/min (equivalent to 2.85 h^{-1}), 50 mL/min H_2 co-flow. Data was taken after 5 h on-stream. Carbon recovery: 95 – 98 %.

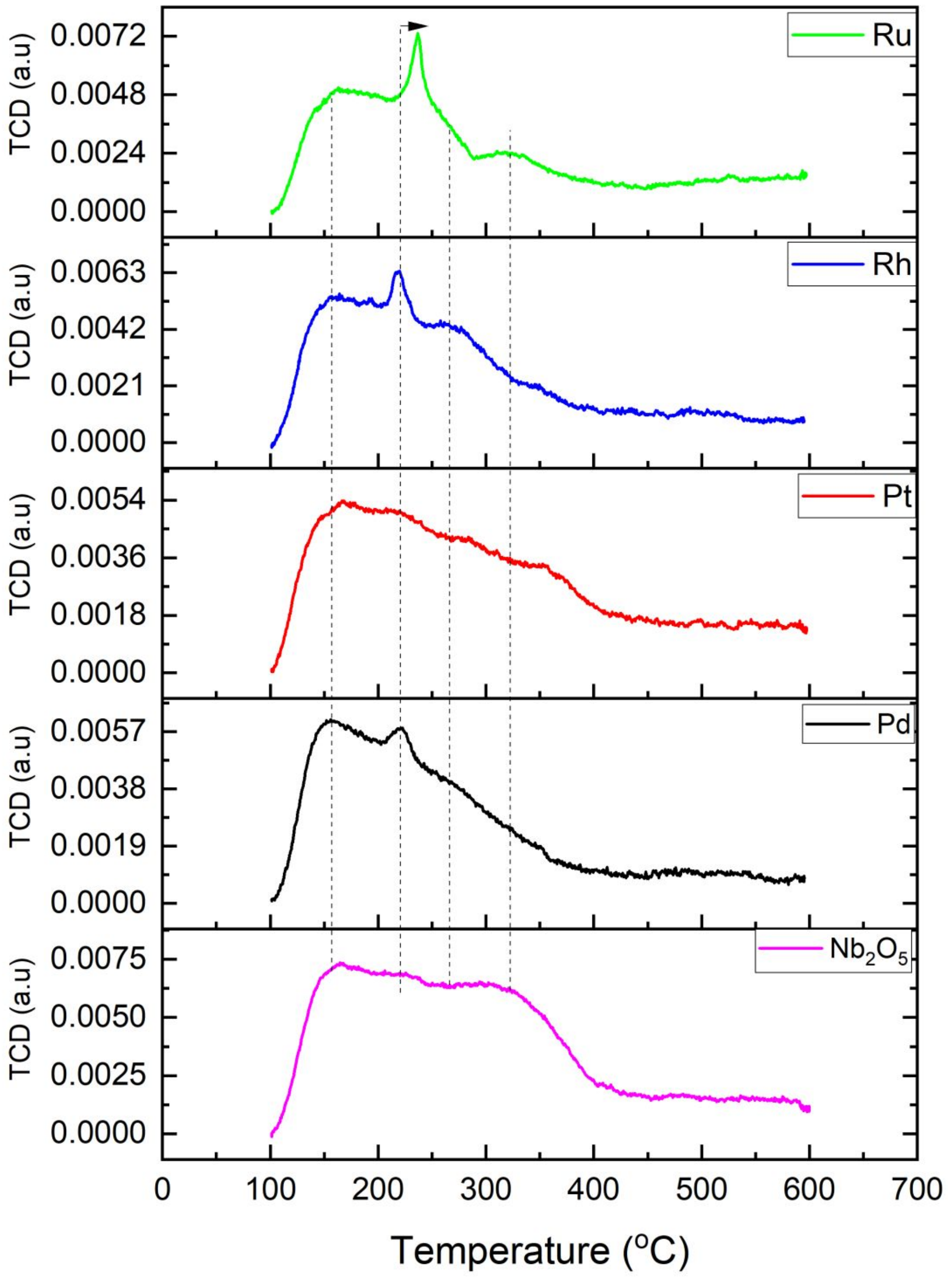

Figure S17. Ammonia temperature programmed desorption profiles for bare niobia and metals supported on niobia.

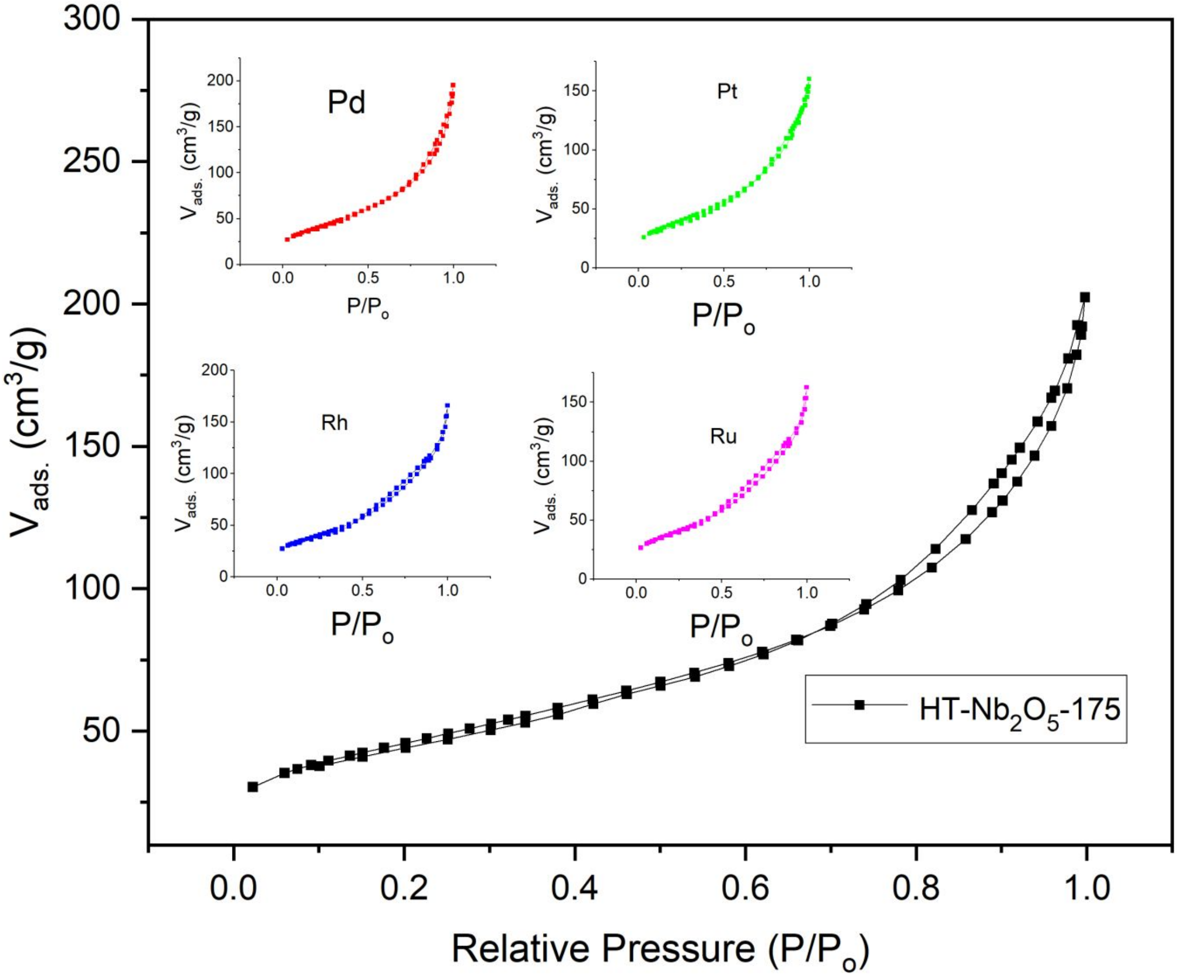

Figure S18. N_2 physisorption isotherms for hydrothermally synthesized Nb_2O_5 and M/Nb_2O_5 (M represents Pd, Pt, Rh, and Ru).

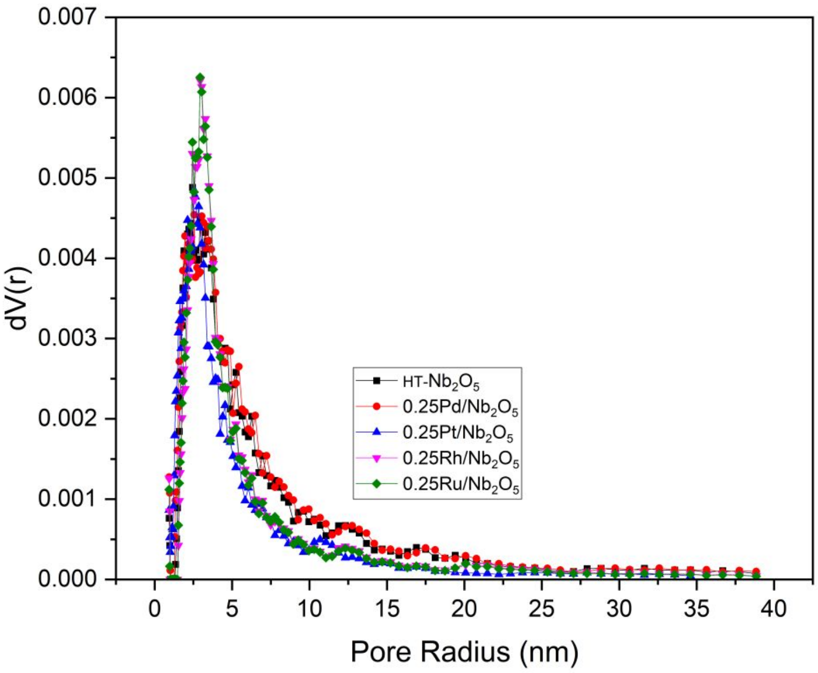

Figure S19. Pore size distribution for hydrothermally synthesized Nb_2O_5 and M/Nb_2O_5 (M represents Pd, Pt, Rh, and Ru) catalysts. Calculation based on non-local density functional theory (NLDFT) model. Pore radius range 2 – 40 nm.

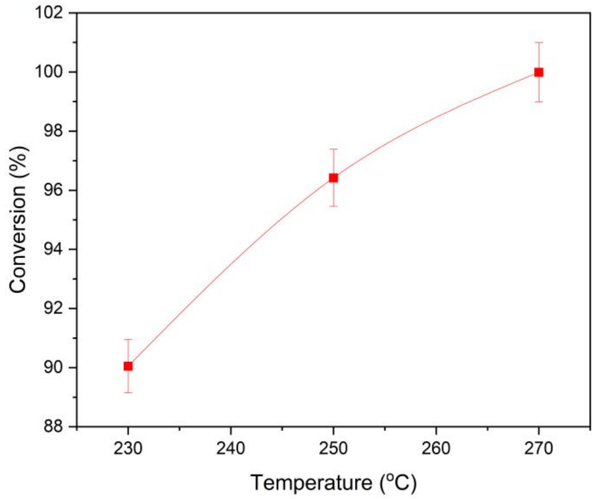

Figure S20. Conversion of model gluconic acid over 0.25%Pd/Nb_2O_5 at 230 – 270 ^oC, 50 bar, and 0.1 mL/min (equivalent to 2.85 h^{-1}). Data were taken after 5 h on-stream. Carbon recovery: 82– 85 %.

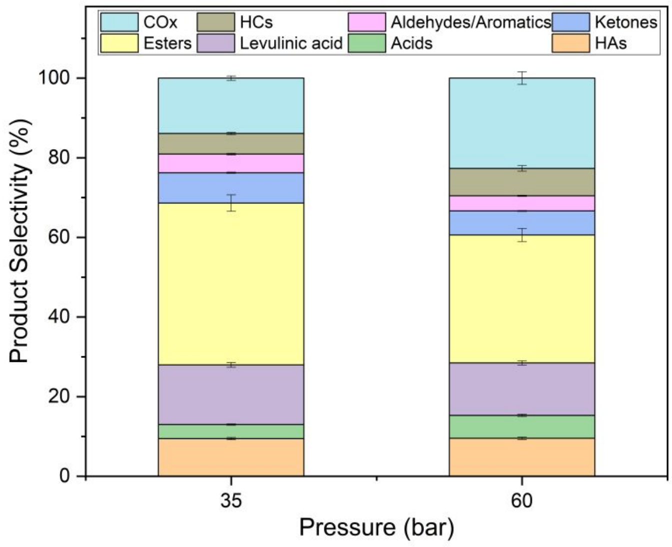

Figure S21. Product selectivity for gluconic acid conversion over 0.25%Pd/Nb_2O_5 as a function of reactor pressure (35 bar and 60 bar) at 250 °C, 0.1 mL/min, and 50 mL/min H_2. Data were taken after 5 h on-stream. Carbon recovery is comparably lower at 35 bar (78%) versus 60 bar (86%).

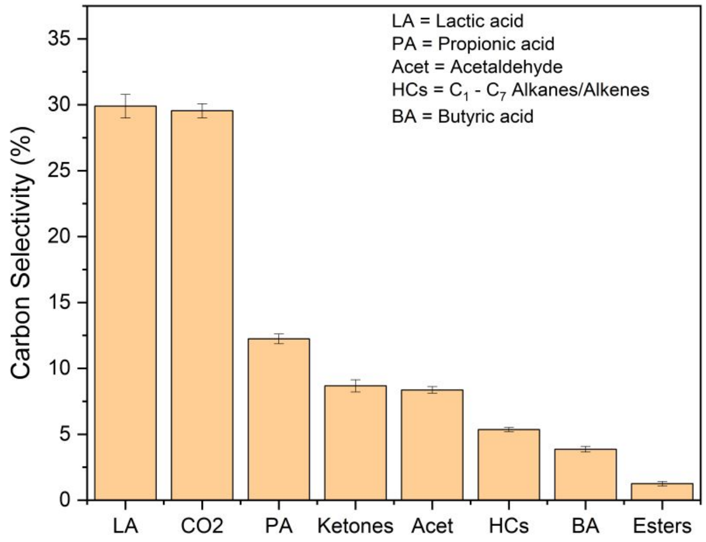

Figure S22. Carbon selectivities for model lactic acid aqueous solution (15 wt%) conversion over 0.25%Pd/Nb_2O_5 at 260 °C, 50 bar, 0.1 mL/min, and 50 mL/min H_2. The conversion of lactic acid at these conditions is ~ 70 %. Data were taken after 4 h on-stream. Carbon recovery: 80%.

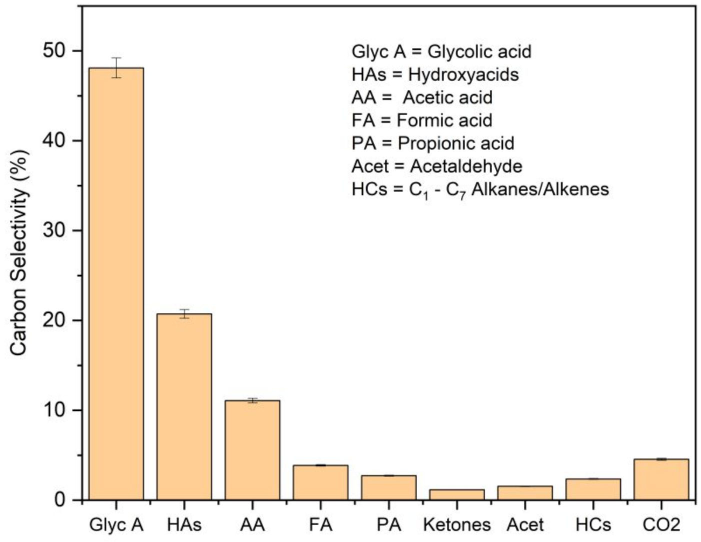

Figure S23. Carbon selectivities for model glycolic acid aqueous solution (13 wt%) conversion over 0.25%Pd/Nb_2O_5 at 260 ^oC, 50 bar, 0.1 mL/min, and 50 mL/min H_2. The conversion of glycolic acid at these conditions is ~ 48 %. Data were taken after 4 h on-stream. HAs constitute up to 95% lactic acid. Carbon recovery: 89%.

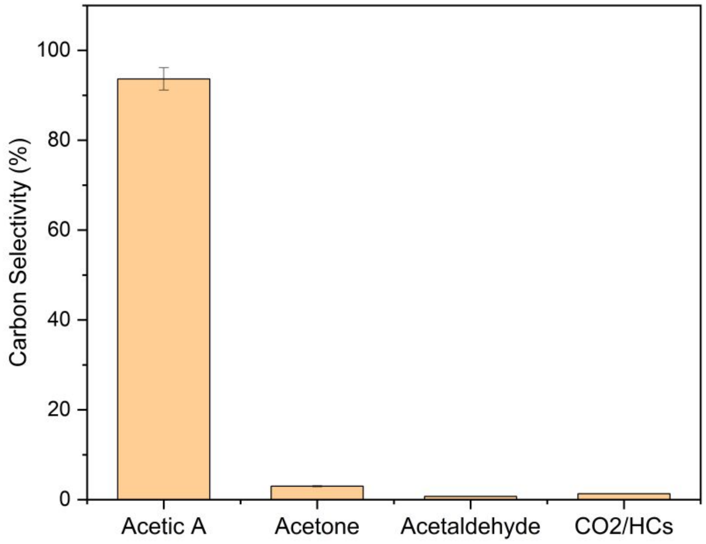

Figure S24. Carbon selectivities for model acetic acid aqueous solution (10 wt%) conversion over 0.25%Pd/Nb_2O_5 at 280 ^oC, 40 bar, 0.1 mL/min and 30 mL/min H_2. Data were taken after 4 h on-stream. Carbon recovery: 98 %.

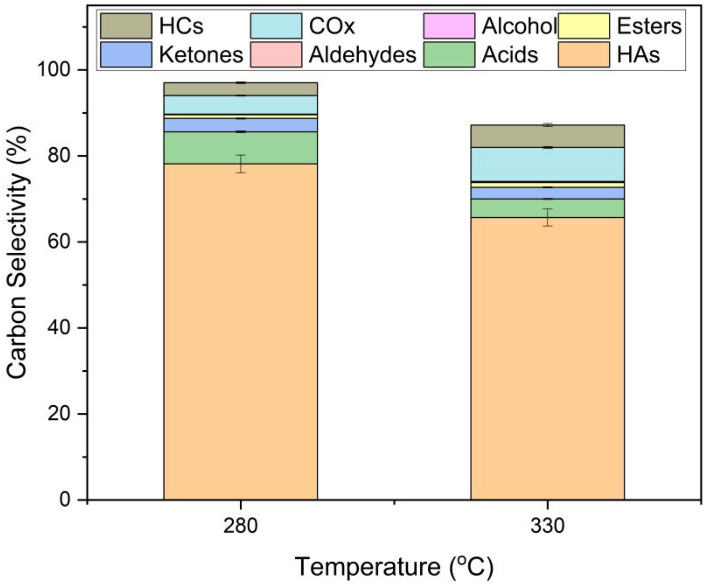

Figure S25. Carbon selectivities in a catalyst-free conversion of BL-derived HA mixture at 60 bar, 0.06 mL/min, and 50 mL/min H_2. Data were taken after 5 h on-stream. Carbon recovery: 77 %.

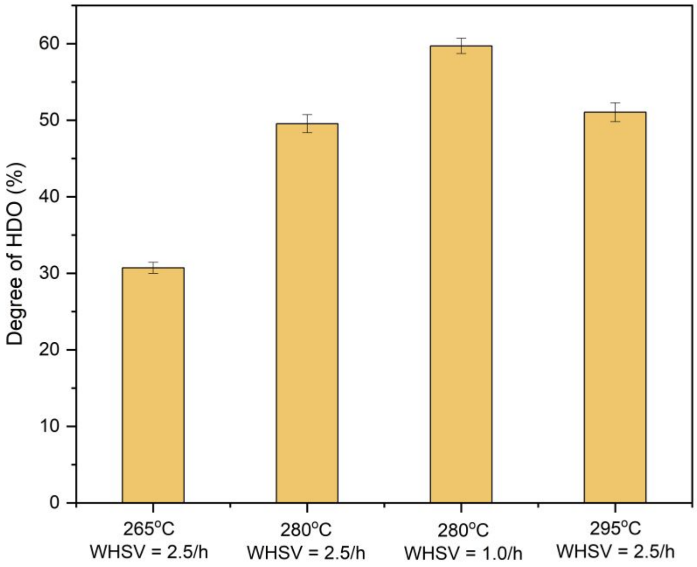

Figure S26. Degree of deoxygenation of kraft BL-derived HAs over 0.25%Pd/Nb_2O_5 at different temperatures and WHSV. The lowest WHSV of 1.0 h^{-1} achieved ~ 60 % deoxygenation.

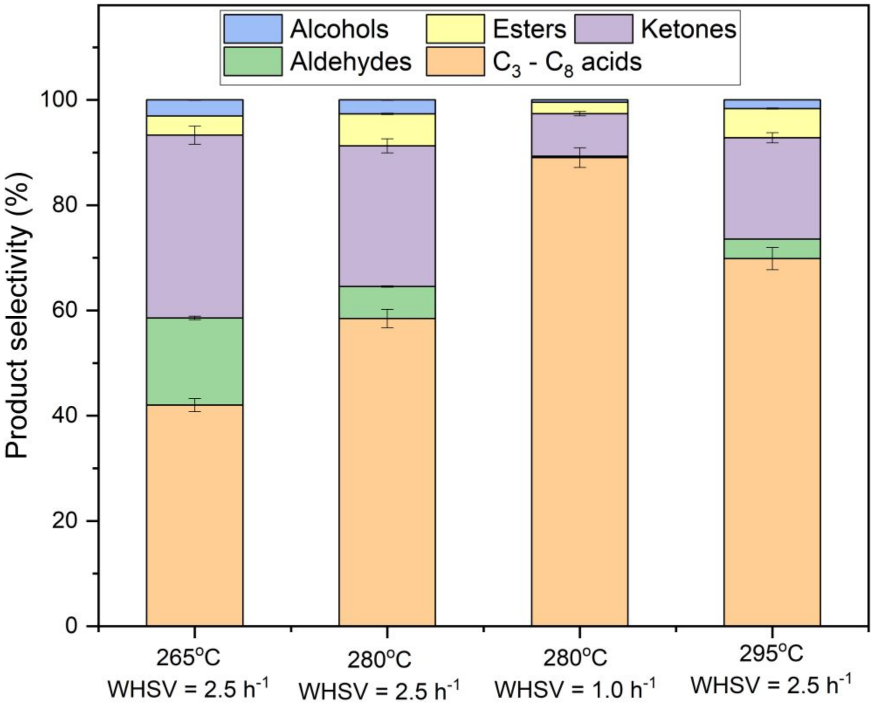

Figure S27. Aqueous phase product selectivities from the catalytic conversion of realistic kraft black-liquor-derived HAs over 0.25%Pd/Nb_2O_5 at different temperatures and WHSV. These are the normalized product selectivities from **Figure 5A** (main text), excluding CO_2 and hydrocarbon products. The selectivity towards C_3-C_8 carboxylic acids reached ~ 90 % (mainly C_3-C_5 carboxylic acids).

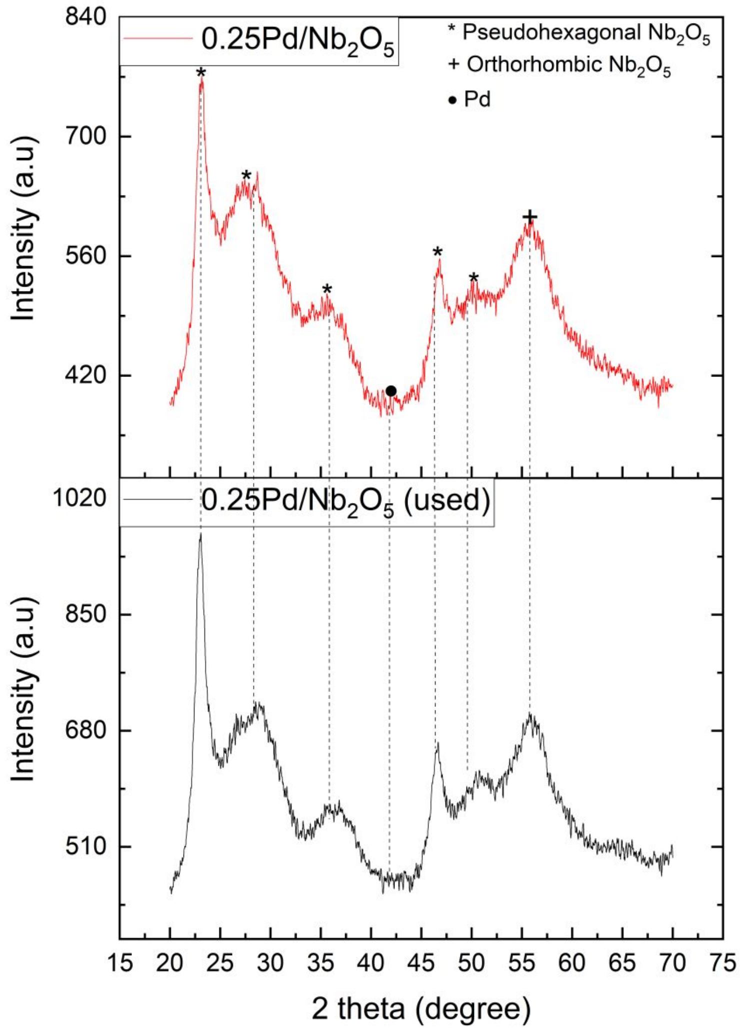

Figure S28. XRD patterns of fresh and used 0.25Pd/Nb_2O_5 showing the presence of different crystallographic phases of Nb_2O_5. The crystallite domain size approximated by the Scherrer equation revealed a slight increase from 12.2 nm (fresh) to 13.6 nm (used).

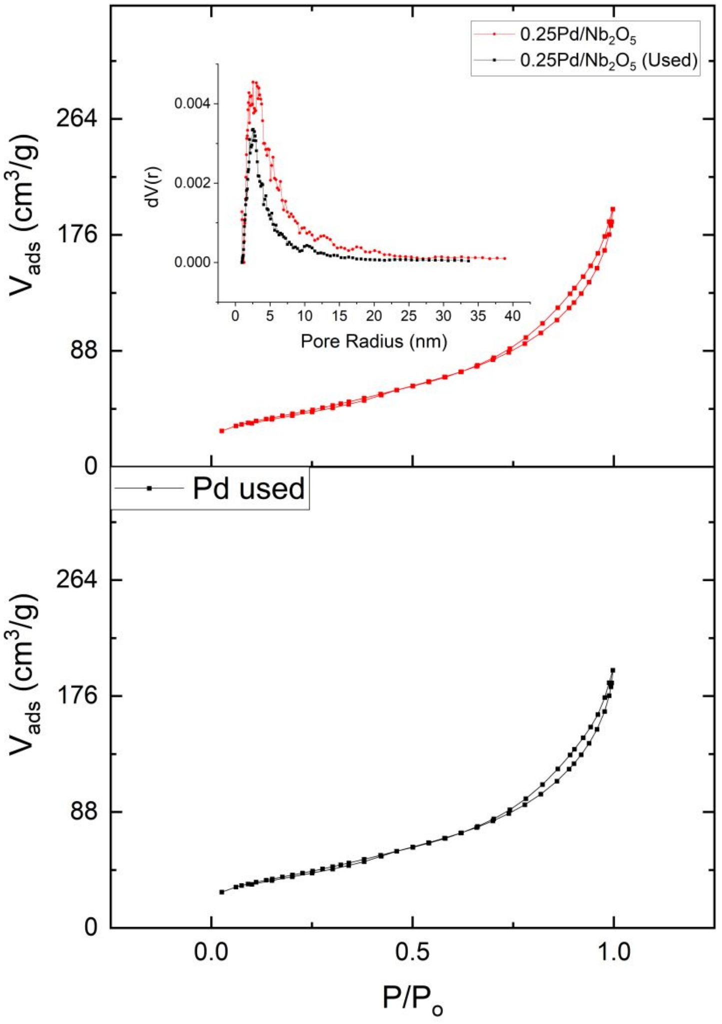

Figure S29. N_2 physisorption isotherm and pore size distribution (inset) for both fresh and used 0.25Pd/Nb_2O_5. A decrease in the BET surface area and a slight size enlargement were observed.

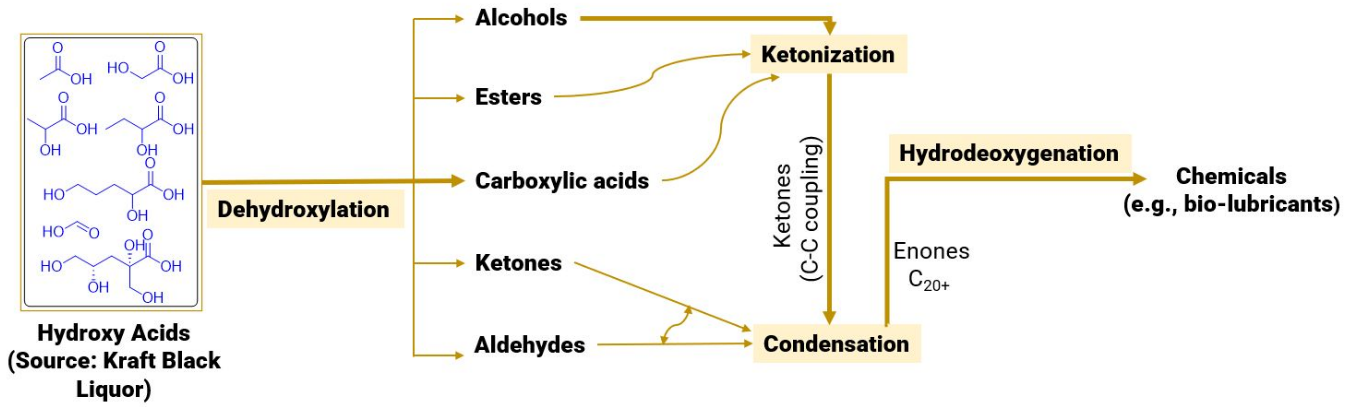

Figure S30. Utilization pathways of products from the hydrodeoxygenation of hydroxy acids derived from kraft black liquor.
